# Exploring Perceived Barriers and Facilitators to Healthier and More Sustainable Dietary Behavior in 27 European Countries: Protocol for a Cross-Sectional Survey

**DOI:** 10.2196/91985

**Published:** 2026-09-30

**Authors:** Anna Maria Murante, Federica Manca, Fabio Consalez, Chiara Chirilli, Luisa Torri, Anant Jani, Agustin Miranda, Eric Verger, Catalina Leon Gonzalez, Maria Piochi, Maria Giovanna Onorati, Susana Campos, Ana Teresa Ferreira Oliveira, Janas Harrington, Valeria Massei, Chiara Ferravante, Tim Smits, Yara Qutteina, Kabi Vanwinkelen, Annemarie Kapteijns, Leonie Barelds-Cramer, Aleksandra Różalska, Aditi Bunker, Till Bärnighausen

**Affiliations:** 1 Centro Interdisciplinare Health Science, Scuola Superiore Sant'Anna, Piazza Martiri della Libertà.33, Pisa, Tuscany, Italy, 39 050883852; 2 Institute of Management, Scuola Superiore Sant'Anna, Pisa, Tuscany, Italy; 3 Sustainable Food Systems, ICLEI European Secretariat, Freiburg im Breisgau, Germany; 4 University of Gastronomic Sciences, Pollenzo, Piedmont, Italy; 5 Heidelberg Institute of Global Health, Heidelberg University, Heidelberg, Baden-Wurttemberg, Germany; 6 Department of Primary Care Health Sciences, University of Oxford, Oxford, England, United Kingdom; 7 MoISA, Univ Montpellier, CIRAD, CIHEAM-IAMM, INRAE, IRD, L’Institut Agro, Montpellier, France; 8 CISAS, Center for Research and Development in Agrifood Systems and Sustainability, Polytechnic University of Viana do Castelo, Viana do Castelo, Portugal; 9 School of Public Health, University College Cork, Cork, Munster, Ireland; 10 Dipartimento Sanità Territoriale, Azienda Unità Sanitaria Locale Toscana Nord Ovest, Pisa, Tuscany, Italy; 11 Azienda Unità Sanitaria Locale Toscana Nord Ovest, Pisa, Tuscany, Italy; 12 Media, Information and Persuasion Lab, Department of Communication Science, KU Leuven, Leuven, Flanders, Belgium; 13 Food & Health Department, Louis Bolk Instituut, Bunnik, Utrecht, The Netherlands; 14 Women's Studies Centre, Department of Cultural Studies, University of Łódź, Lodz, Łódź Voivodeship, Poland

**Keywords:** food behaviors, health, sustainability, food systems, vulnerable groups, health inequality, system policy

## Abstract

**Background:**

Food systems strongly affect human and environmental health. Across Europe, they often generate negative public health and environmental outcomes and contribute to inequalities in access to healthy, sustainable food. Addressing these challenges requires understanding the complex dynamics of the interaction of individual behavior within food systems, which involves a combination of internal mechanisms and external influences.

**Objective:**

This study, part of the FEAST project (Food Systems That Support Transitions to Healthy and Sustainable Diets), aims to increase understanding of how micro-, meso-, and macro-level factors shape Europeans’ food preferences, purchasing, and consumption behaviors. It also explores differences across European regions and population subgroups.

**Methods:**

A cross-sectional survey was conducted among 27,000 adults in 27 European countries across the 5 FEAST food regions (Anglo-Saxon, Central European, Scandinavian, Southern European, and Eastern European). In addition to the vulnerable groups (older adults; low-income or low-education people and those with health or disability conditions; families with minors, women, gender-diverse people, etc) that could be captured in the main survey, an additional sample of 400 vulnerable individuals from the Down syndrome (DS) community (including carers of people with DS) was expected to be involved in 5 European countries (Ireland, Italy, Portugal, Romania, and Scotland). To achieve the target of 1000 participants per country, the general population was recruited through a market-insights platform with extensive European coverage. Individuals with DS and their caregivers were recruited through national DS associations. Data were collected using an 88-item questionnaire covering dietary patterns, food-purchasing behavior, drivers and barriers to healthy and sustainable eating, perceived dietary sustainability, opinions on food policy options, and sociodemographic characteristics. The same questionnaire was administered to caregivers, whereas individuals with DS completed an adapted version addressing eating habits, food appearance, and food neophobia in an accessible format. Statistical analyses are being conducted to identify determinants of healthy and sustainable diets.

**Results:**

A total of 27,417 questionnaires were collected from the general population across 27 countries, together with 82 questionnaires from people with DS and 242 from caregivers. Expected findings will describe Europeans’ food consumption habits, preferences, and purchasing behaviors, including those of vulnerable groups, and assess their alignment with planetary health diet principles. The study will also identify key barriers and facilitators to adopting healthier and more sustainable diets, as well as sources of nutritional information and attitudes toward food-related policies.

**Conclusions:**

The findings will support our understanding of individual dietary habits and the development of multilevel strategies to promote healthier and more sustainable diets in these population subgroups across Europe. They will also provide insights into interactions between consumers and the food system, highlighting barriers and facilitators that influence dietary choices.

## Introduction

### Background

Food systems have a significant impact on the opportunities for improving or degrading human and environmental health. Europe’s current food systems, unfortunately, largely degrade both [1]. Food systems are accountable for substantial environmental degradation, comprising 26% of global greenhouse gas emissions, consuming 50% of global habitable land use, using 70% of freshwater resources, contributing to 78% of eutrophication, and driving 60% of biodiversity loss [2]. This impact extends beyond environmental concerns. Current food systems play a critical role in perpetuating preventable diseases, both in Europe and globally, resulting in direct and indirect costs to society, further undermining health systems and the economy.

Moreover, food systems have contributed to the exacerbation of health inequalities across Europe, perpetuating disparities in access to healthy and sustainable food [2]. This criticality can be exacerbated in vulnerable groups such as older adults, women, gender-diverse people, households with minors, people with low income, people in poor health, or individuals with disabilities.

The intricate and dynamic nature of food systems presents a significant challenge when it comes to implementing strategies to promote fairness, health, and sustainability. Like any complex systems, food systems are shaped by a combination of internal mechanisms and external influences. A multitude of actors, covering various spatial and temporal dimensions, contribute to this complexity with their diverse values, priorities, and practices. Consequently, this diversity can lead to conflicting interests, tensions, and unresolved issues, resulting in fundamental uncertainties on their future evolution [1].

In this context, our study aims to make 2 key contributions. First, it aims to generate new insights about individual-level dietary behaviors as well as factors perceived as influencing these behaviors in order to inform micro-, meso-, and macrolevel approaches that can support different subgroups in Europe to adopt and adhere to healthier and more sustainable dietary behaviors. Second, the results aim to inform policies that will support Europe’s net zero targets and the creation of “a favourable food environment able to facilitate healthy and sustainable dietary choices, which will benefit consumers’ health and quality of life and reduce health-related costs to society” [3].

This protocol outlines a research study that will explore the relationship between micro-, meso-, and macrolevel factors perceived to influence dietary behavior in diverse contexts and different population subgroups across Europe as well as individual-level food preferences and purchasing and consumption behavior (eg, social, cultural, marketing, food distribution, regulatory, political, socioeconomic, etc). It also aims to consider diverse food environments spanning urban, suburban, and rural areas across Europe and to understand the unique dynamics associated with dietary behavior for different population subgroups, including those with different types of vulnerabilities.

This study has the potential to make an important contribution to the current landscape of research on dietary behaviors within Europe, which is often characterized by various limitations and disparities. For example, existing studies are often confined to a selection of a few European countries, using different populations, survey methodologies, and tools [4-6]. Traditional nutritional assessment techniques (eg, 24-hour dietary recalls, food frequency questionnaires, and food diaries) are widely used in dietary research, but they often are not adequate in capturing the multifaceted nature of dietary behaviors and preferences [7]. Important aspects such as behavioral patterns, communication dynamics around food products, and their implications for fostering healthier and more sustainable dietary behaviors are often overlooked [8,9]. This lack of standardization and methodological issues makes the use and harmonization of data across studies difficult, making it challenging to draw comprehensive and comparable conclusions across different contexts and different population subgroups [10,11]. The unique and comprehensive design of our study directly aims to address these issues.

### Research Objective

This study proposes a comprehensive approach to mapping and monitoring food consumption behaviors related to healthy and sustainable dietary choices across Europe, with a specific focus on identifying the perceived barriers and facilitators associated with individuals’ dietary habits. Aggregating and synthesizing these individual-level insights will inform food system stakeholders about the relationship between micro-, meso-, and macrolevel factors and individuals’ food preferences and purchasing and consumption behavior to support the identification of priorities in food system policy and practice improvements to stem unhealthy and unsustainable food choices. To achieve our aim, the study will also delve into individual food habits to identify “Natural European Dietary Segments” (NEDS) as an alternative to traditional segments (children vs adults, women vs men, low income vs high income, etc). The NEDS will represent the composition of homogeneous groups based on how healthy and sustainable their eating habits are. Their purchasing behaviors and consumption habits will also be explored to understand and shed light on the dynamics supported or limited by specific facilitators and barriers arising from the individual, commercial, production, or policy spheres that influence the individuals’ dietary choices.

## Methods

### Overview

This study is part of the FEAST (Food Systems That Support Transitions to Healthy and Sustainable Diets) project, funded by the European Union (EU) HORIZON Europe Framework, under the call HORIZON-CL6-2021-FARM2FORK-01‐15 [1,12] and cofunded by UK Research and Innovation and Swiss Research Foundation. It involves a collaborative consortium that uses the expertise of 35 partner institutions across 15 European countries representing academia, the public sector, civil society organizations, and the private sector. FEAST aims to make it easy for every person in Europe to eat a delicious, healthier, and more sustainable diet. This aim holds the potential to benefit a multitude of stakeholders, encompassing the health of populations, environmental preservation, support for food producers and industry, and the alleviation of societal costs linked to poor diets and suboptimal food systems. Crucially, the project recognizes the importance of addressing behavioral dimensions within food system solutions, a component often overlooked. FEAST’s targeted activities engage stakeholders across the 3 levels of food systems: macro, meso, and micro. At the microlevel, the focus extends to people in Europe, diverse population subgroups, and nongovernmental consumer and patient organizations. One means by which people in Europe are involved in FEAST is through a large-scale study based on a cross-sectional survey, which is presented here as the main component of this study.

### Study Design and Participants

A quantitative cross-sectional research design to collect quantitative primary data has been used in this survey study [13]. Respondents from 27 European countries (Figure 1), across FEAST’s 5 food regions (Anglo-Saxon, Central European, Scandinavian, Southern Europe, and Eastern Europe) defined by food culture, agricultural production systems (mostly small, medium, or large farms), and welfare systems (Beveridge or Bismarckian) (1), have been enrolled. We expected to involve a stratified sample of 27,000 adult (aged 18+ years) individuals, which would yield 1000 participants per country in 27 European countries.

In addition to vulnerable groups (older adults, women, gender-diverse people, children, people with low income, and people in poor health) that could be captured in the main survey, an opportunistic sample of about 400 persons with Down syndrome (DS) and their carers are expected to be enrolled from 5 countries. Individuals with DS represent a population subgroup with a food-related vulnerability due to the dysfunctions and the physical and psychological characteristics associated with the condition (hypotonia, small oral cavity, enlarged tongue, and poor sucking reflex), which can impact feeding. Due to these physical characteristics, individuals with DS are at high risk and have low resilience in accessing food, and their vulnerability may also be increased by parental food choice behaviors. The eating behavior of young people with DS and parenting styles regarding eating practices are important aspects to focus on because of their potential implications for health status, including obesity, malabsorption, and type 2 diabetes [14].

**Figure 1.**
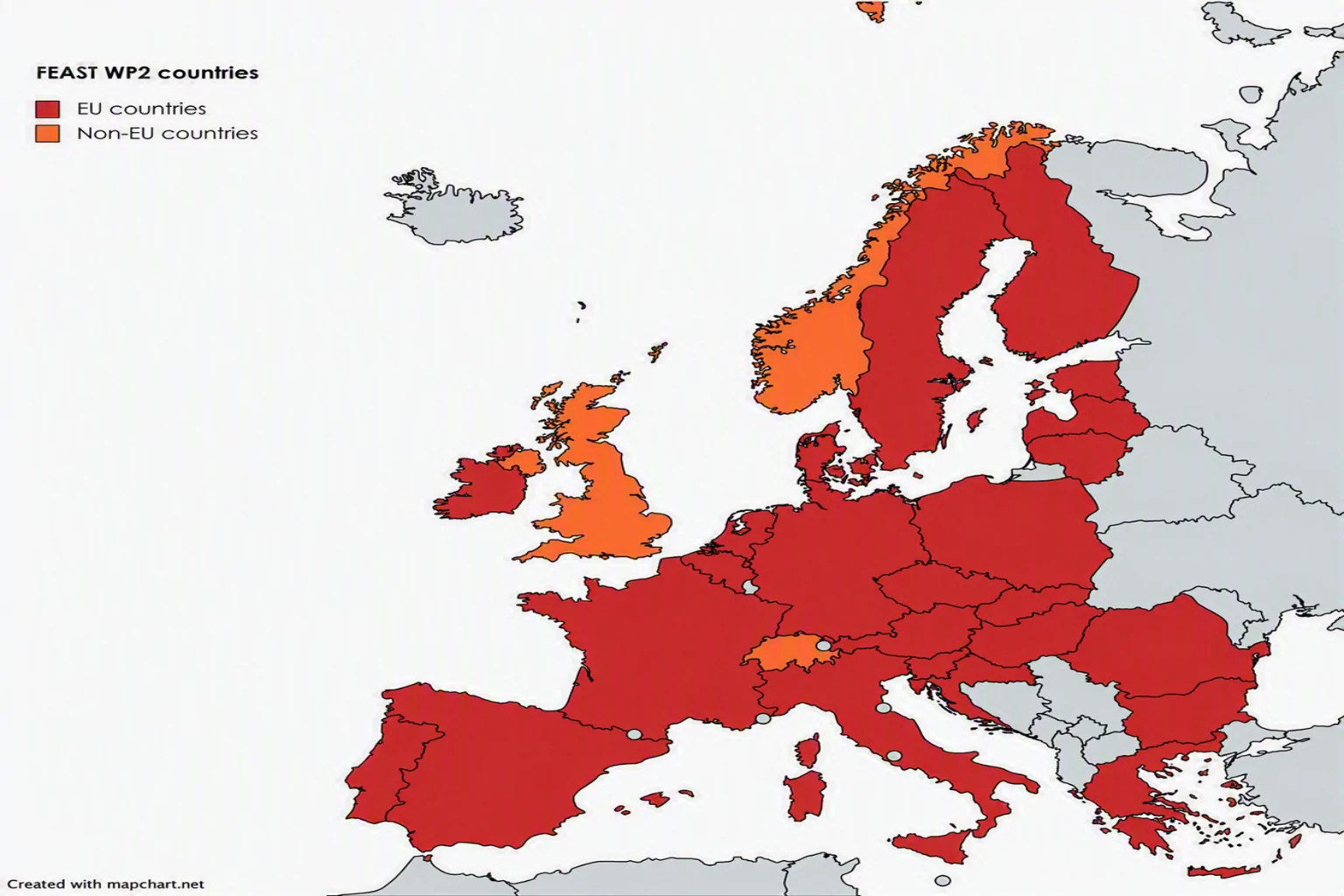
Target countries included in the study across Europe: Austria, Belgium, Bulgaria, Croatia, Czechia, Denmark, Estonia, Finland, France, Germany, Greece, Hungary, Ireland*, Italy*, Latvia, Lithuania, Netherlands, Norway, Poland, Portugal*, Romania*, Slovenia, Slovakia, Spain, Sweden, Switzerland, and United Kingdom* (*in these countries, we also involved the Down syndrome community). EU: European Union; FEAST project: Food Systems That Support Transitions to Healthy and Sustainable Diets.

### Sample Design

The first survey, designed for the general adult population in Europe, aims to reach a population sample with characteristics that would be coherent with the demographic composition of the populations living in 27 countries within the 5 FEAST food regions. To this end, a stratified sample design has guided the participants’ enrollment (Table 1). Specifically, we would recruit 1000 adults who will be representative of the target population for age, sex, and education (defined by the International Standard Classification of Education [ISCED]–2011 classification system) within each selected country [15]. The sample strata were designed using Eurostat demographic data, which also served as the basis for calculating sample weights. These weights were then applied to correct for under- or overresponse and to approximate the characteristics of the general European population. Unfortunately, Eurostat data do not include information on gender distribution and do not report statistics on the level of educational attainment for adults older than 75 years [16]. However, we have collected these characteristics by including education level and gender items in the questionnaire to consider these variables in the analyses.

The second survey, designed to explore the food behaviors of individuals with DS and their relatives, used an opportunistic sampling approach at the country level, given its exploratory nature. This choice was necessary because, even if country-level sociodemographic data had been available to inform a stratified design, achieving nationally representative samples would have remained unfeasible due to structural barriers limiting survey accessibility for people with DS. Overall, we consider the DS individual and carer surveys exploratory works to bring attention to food-related vulnerabilities for people with DS and their relatives.

**Table 1.** Sampling stratification strategy based on sex, age group, and educational attainment level (according to ISCED^a^ 2011) for each country included in the study (N=1000 individuals per country). Quotas were defined to match national population distributions based on Eurostat data.

Sex and age group, years		Educational attainment level (ISCED-2011)
Female		
	18-24	ED^b^ 0‐2; ED 3‐4; ED 5‐8
	25-34	ED 0‐2; ED 3‐4; ED 5‐8
	35-44	ED 0‐2; ED 3‐4; ED 5‐8
	45-54	ED 0‐2; ED 3‐4; ED 5‐8
	55-74	ED 0‐2; ED 3‐4; ED 5‐8
	75-84	N/D^c^
	85 and older	N/D
Male		
	18-24	ED 0‐2; ED 3‐4; ED 5‐8
	25-34	ED 0‐2; ED 3‐4; ED 5‐8
	35-44	ED 0‐2; ED 3‐4; ED 5‐8
	45-54	ED 0‐2; ED 3‐4; ED 5‐8
	55-74	ED 0‐2; ED 3‐4; ED 5‐8
	75-84	N/D
	85 and older	N/D

a ISCED: International Standard Classification of Education.

b ED: education.

c N/D: not determined.

### Recruitment

To achieve our target of 27,000 participants to involve in the first large survey, we have relied on the services of a market-insights gathering platform, Cint, with an extensive reach of panel members across Europe. Acting as panel aggregators, market-research panels enable researchers to potentially reach large samples of people across the world, gathering data from multiple countries in very short periods of time, including granting access to hard-to-reach demographic groups, thus favoring a higher representativeness of the sample [17]. Although this recruitment strategy has the advantage of ensuring broad geographic coverage and reaching a population with similar demographic characteristics to the target group, it has its disadvantages. The final sample may be affected by selection biases and leave some vulnerable groups underrepresented with some effect on the generalization of results. For transparency, researchers will pay closer attention to ensuring that findings are consistent with those reported in existing literature when they are available. Any misalignment between the data collected and the population data will be declared in the research outputs. For example, young people with lower education and older adult people are underrepresented in our sample due to the difficulties of reaching them.

The platform has shared the web survey with panel members. Unfortunately, it is not possible to know what the main barriers to participation were (availability, literacy, or digital limitations), or whether strategies useful for facilitating participation were implemented because the panel service did not provide details on this point. This leaves a gap in our understanding of the survey administration process as well as the drivers or barriers to participation.

To achieve representativeness of the sample, recruitment was limited by quotas developed on the basis of the demographic distribution of each country by sex, age, and educational attainment level, as defined by the sampling design (Table 1). Once quotas for a specific demographic group have been achieved, participants within that quota are no longer able to complete the survey, thus ensuring that all quotas are filled and representativeness is achieved. Data collection was deemed to be complete when the goal of 1000 complete responses is reached in each country.

The recruitment of members of the DS community (individuals with DS and their carers) has involved 4 national DS associations in Ireland, Italy, Romania, and Scotland, and a local association in Portugal. Each national association would adopt strategies appropriate to their organization to disseminate the web questionnaire (eg, by sharing the link with local offices and through them to all members, or by publishing it on their website and social media). The Portuguese branch has shared the survey with other local delegations and has involved DS members with the involvement of educators during the daily activities organized in the local facilities. Information sheets with a simple communication style, completed with an appendix on the data treatment policy to be read with the support of a carer if necessary, were used to introduce the study,

### Questionnaire for the General Population

To explore the dietary habits of adults in Europe and the drivers and barriers that they face in eating healthier and more sustainable diets, we designed a cross-sectional web survey, which consists of an 88-item questionnaire aimed at identifying items able to support the achievement of the main FEAST project’s objectives [1]:

1. To identify, understand, and measure the perceived barriers and facilitators to healthy and sustainable dietary behavior of different groups (particularly vulnerable groups in Europe), accounting for geographical, socioeconomic, behavioral, gender, and cultural differences.
2. To cocreate (ideation, design, and testing) innovative and effective tools, programs, and strategies, including social innovations, in collaboration with key stakeholders in Europe that will enable consumers to make informed food choices that promote the self-management of healthier and more sustainable dietary behaviors and lifestyles.
3. To empower individuals to lead healthier lives by adopting healthier and more sustainable dietary behaviors, choices, and lifestyles through evidence-based strategies and tools that address all food system actors at the level of member states, the EU, and the wider international community.
4. To boost the adoption of food and health policy interventions that aim to drive the transition to healthier and more sustainable diets by all stakeholders within the food system by using co-design and scientific testing of communication strategies, and associated monitoring approaches, that could be used by policymakers.

The questionnaire development followed a 3-step procedure. First, a review of the scientific and gray literature was conducted to identify factors relevant to dietary behaviors and factors influencing these behaviors. The review aimed to answer the following research question: “What is currently known about consumers’ attitudes, behaviors, drivers, and barriers towards healthy and sustainable diets?” The second step of the development process consisted of 2 consecutive rounds of voting, where each team member could rank selected items from the review conducted to identify factors relevant to dietary behaviors and factors influencing these behaviors. Finally, the questionnaire was developed and translated into the 24 languages spoken across target countries. The translation process was qualitatively validated by members of the FEAST consortium who knew the language and culture of the country where the survey was to be addressed for the 15 countries represented in the consortium. They compared the translated questionnaire provided by Cint in their national language with the English version and proposed revisions where necessary. The evidence obtained on questionnaire validity revealed no cross-country differences suggestive of translation bias. The questionnaire underwent a full validation process upon completion of data collection, confirming the preliminary evidence of validity and reliability observed in the first 1350 collected questionnaires. The full questionnaire consisting of 88 items divided into 8 sections (Figure 2) can be seen in Multimedia Appendix 1. In the following paragraphs, we provide a brief overview of each subsection.

**Figure 2.**
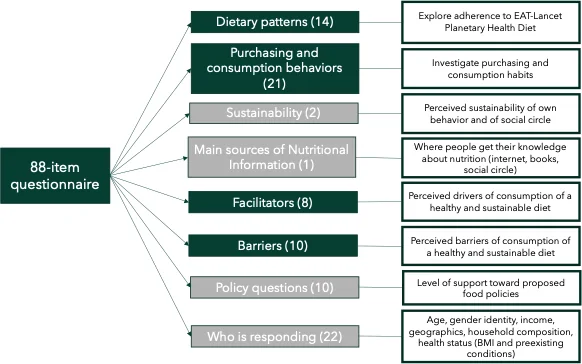
Structure of the questionnaire: 88 items across 8 sections, number of items per section, and item response formats used (estimated administration time = 22 minutes).

We developed a simplified food frequency questionnaire to assess dietary behaviors across Europe. It measures how often food and nutrient categories are consumed, but not how much, and can reflect general dietary tendencies or patterns indicating how closely individuals align with the EAT-Lancet Planetary Health Diet principles [18]. The planetary health diet provides guidelines to promote the consumption of a healthy and sustainable diet by strongly reducing the consumption of animal-sourced foods and promoting the consumption of plant-sourced foods. Hence, we explore the consumption of the 14 food and nutrient categories (whole grains, tubers and starches, vegetables, fruits, dairy, red meat, poultry, eggs, fish and shellfish, legumes, nuts, all plant oils, unsaturated oils, saturated or animal fats, and added sugars) from the planetary health diet. The focus on added sugar, rather than on other nutrients of concern such as sodium, saturated fatty acids, or fiber, reflects the strong policy attention that sugar-sweetened beverages and sugar taxation were receiving at the time this study was designed.

For each food group and nutrient, 7 levels of frequency of consumption were measured: never, 1-3 times per month, once per week, from 2 to 3 times per week, from 4 to 6 times per week, daily (once a day), and 2 or more times daily. This scale measures the frequency of consumption of food and nutrient categories, but it is important to note that it was not designed to capture habitual intakes, nor was it designed to; it serves as a proxy indicator about the individual behaviors consistent with planetary health diet principles (eat mostly plant-based food, reduce animal-source food, choose healthy fats, limit added sugars and refined grains, etc). This is consistent with the survey’s goal of understanding general dietary behavior and factors that are associated with it.

To make the evaluation of dietary patterns more effective, collected data have been used to develop the EAT-Lancet Consumption Frequency Index (ELFI) score to assess the food group and nutrient consumption of Europeans based on planetary health diet principles. A quantile-based scoring system is applied, in which higher scores reflect more frequent consumption of recommended foods and lower consumption of foods to be limited. The total score ranges from 0 to 42, with higher values indicating greater alignment to planetary health diet principles. Further details on the scoring procedure are described elsewhere [19]. The instrument demonstrated strong internal consistency (Cronbach α=0.83) and a stable bidimensional structure confirmed through exploratory and confirmatory factor analyses. In this sense, food and nutrient categories are categorized into 2 subscales: “foods to encourage” (eg, fruits, vegetables, legumes, whole grains, nuts, and fish) and “foods to balance and to limit” (eg, red meat, dairy, eggs, added sugars, and animal fats). Convergent validity was supported through correlation with a validated dietary index based on 24-hour recalls, and further evidence of validity was provided through expected associations with dietary intake, sociodemographic variables, nutritional health, and environmental impact indicators [20]. The latter insights support the use of ELFI as a good proxy measure of healthy and sustainable food behaviors.

We also set out to investigate purchasing and consumption behaviors of respondents across Europe by addressing behaviors specifically related to habits of cooking and food purchasing within the household, as well as perceived factors driving the consumption and purchase of foods. Questions on purchasing behavior were developed to address whether consumers make their choices based on individual factors (“How often do you consider the following elements when purchasing/consuming food?”), such as cost value, overall liking of the product, and religious beliefs; elements related to food item per se, such as nutritional value and seasonality; convenience, whether tied to the ease of preparation, as well as to the availability where the item is purchased; the packaging, both in size and appearance, as well as in the labeling; and, finally, related to environmental impact, both as the knowledge of the impact and through the availability of certifications to attest to ethical and sustainable production [20-22]. The extent to which individuals considered these items during purchasing choices is investigated through 5 possible levels of frequency: never, rarely, sometimes, often, and always. One section of the questionnaire is dedicated to exploring respondents’ perceptions of the sustainability of their dietary behaviors and those of their social circles.

To investigate factors influencing the individual adoption of healthy and sustainable dietary practices, the questionnaire includes questions about both perceived facilitators and barriers (“To what extent do these elements support you to eat a healthy and sustainable diet?” and “To what extent do these elements limit you to eat a healthy and sustainable diet?”). Eight items have been designed to gauge the extent to which various elements are perceived to support individuals in adhering to a healthy and sustainable diet. These elements include personal knowledge about healthy and sustainable diets, the availability of practical tools and resources, access to healthy food choices, social expectations, external pressures, the influence of peers’ eating habits, personal health goals, and access to information on sustainable food production. Conversely, the barriers section comprises 10 questions aimed at identifying factors that individuals perceive may hinder them from maintaining a healthy and sustainable diet. These barriers encompass issues such as irregular working hours, busy lifestyles, reluctance to give up preferred foods, challenges associated with unfamiliar or unusual foods, affordability concerns, difficulties in meal preparation, lack of willpower and knowledge, the influence of peers’ unhealthy eating habits, and limited availability of healthy food options in proximity to one’s residence or workplace. The perceived role of these items on the consumption of a healthy and sustainable diet is investigated through a 5-point Likert scale: 1—Not at all; 2, 3, 4, 5—Completely.

The questionnaire also elicits respondents’ opinions on various food policies. Through 10 statements, participants are asked to express their support or opposition to each proposed policy. The policies cover a range of measures aimed at promoting healthier and more sustainable food consumption practices. These policies include increasing taxes on products with higher added sugar, implementing a 0% value-added tax (VAT) on fruits and vegetables, banning price discounts for unhealthy food and beverages, prohibiting the marketing of unhealthy products on various media platforms (eg, social media, television, and magazines), and restricting their placement at supermarket checkouts. Additionally, policies addressing the marketing of unhealthy foods to children, outdoor advertisements for such products, sales of energy drinks to minors, taxes on red meat production, and the inclusion of environmental sustainability labels on packaged foods are presented. Participants are provided with response options to indicate their level of support or opposition to each policy, along with options to refrain from responding or to indicate uncertainty on the options [23].

For the last 3 questionnaire components (purchasing and consumption behaviors, facilitators and barriers for healthy and sustainable eating, and attitudes toward food policies), psychometric validation analyses were conducted. The Drivers and Barriers section demonstrated high internal consistency and a stable 2-factor structure, and the Purchasing and Consumption section showed good reliability and a clear 3-factor structure. The items about the attitudes toward food policies included in the Policy section demonstrated good internal consistency and a largely unidimensional section. Across modules, factor sections were supported by confirmatory factor analyses and remained stable across 24 European language groups, indicating cross-country comparability. In addition, nomological validity was supported through theoretically consistent associations between questionnaire constructs and dietary behavior.

A further section explores the main nutritional information sources, including media (both traditional and online), family, friends and/or peers, health professionals and/or health authorities, and other relevant sources [24].

To conclude, the questionnaire collects information about respondents’ age, level of education, gender identity, principal occupational status, household net yearly income, perception of household income sufficiency, household composition, country of birth, country of birth of parents, country of residence and duration of stay, description of residential area, residential zip code, description of work or study location, work or study zip code, preexisting health conditions, height, and weight.

### Questionnaire for Persons With DS

The research team prepared a questionnaire starting from the literature and translated the contents to 4 languages (English, Italian, Portuguese, and Romanian). Each national DS association approved the contents of the questionnaire as easy to complete. The questionnaire for persons with DS consists of 71 items. It was developed to explore the existing dietary patterns of people with DS and to understand how specific factors related to their physical and psychological status may play a role in their food choices. The research team used validated scales from the literature to measure the role of (1) sensory appeal, (2) eating behavior and feelings, (3) food texture preference, (4) food neophobia, and (5) proenvironmental behavior. Ad hoc questions—mostly aligned with the food questionnaire scale used for the adult population—were used to explore frequency of consumption of 16 food categories. An additional 4 items were included to collect information on respondent characteristics (country, age, gender, and where they usually eat their main meals). Items are proposed with the support of pictures that reproduce the item content to make it easier for people with DS to understand the questions. Pictures were generated using the Ideogram software (Ideogram, Inc). The 7 main dimensions of the DS questionnaire are described in the following paragraphs (Multimedia Appendix 1).

It has been observed that people with DS eat too little fruit and vegetables, whole grain cereals, and dairy products, and too much meat and sweets. To support their consumption of a healthier and more sustainable diet, it is sufficient to follow the recommendation proposed for the general population, taking care to adapt the diet to any specific health complication people with DS may face (such as type 2 diabetes) [25]. For this reason, we developed a food frequency scale including the 14 food and nutrient categories proposed by the EAT-Lancet Planetary Health Diet and also used in the questionnaire proposed to carers, plus 2 additional items on consumption of fizzy drinks and fast food. Responses measure frequency of consumption from never to 2 or more times per day.

The literature also highlights that sensory properties such as taste, texture, appearance, and smell could significantly influence food acceptance, particularly in vulnerable groups such as people with DS. Developing new food products for people with DS presents unique challenges, especially given the impact of sensory factors on food acceptance and rejection, as well as on more challenging eating behaviors (eg, food refusal or selective eating) [26]. The DS questionnaire includes a scale of items exploring what people with DS look for in food. It specifically collects information on the potential contribution of smell, appearance, texture, and taste to the food experience of people with DS. Respondents can give an answer between “Not important,” “Maybe,” and “Important.” This scale is an adaptation of the “sensory appeal” scale [27]; the adaptation makes it easier for people with DS to respond. We have also introduced pictures showing a thumbs up or thumbs down depending on the answer available.

An adaptation of the “Child Three-Factor Eating Questionnaire” [28] was used to investigate the eating behaviors of individuals with DS. Respondents answered 17 questions. For items 1‐16, they were asked to select an answer between “No,” “Maybe,” and “Yes,” with pictures of a thumbs up or thumbs down to indicate their responses. For item 17, respondents answered the question, “How often do you feel hungry?” by choosing between the options “Only at mealtimes,” “In between meals,” and “All the time.”

Individuals with DS often face challenges related to chewing and food texture. To understand these challenges, an adapted version of the “Child Food Texture Preference Questionnaire” [29] was used to investigate food texture preferences. Respondents were shown 17 pairs of food images and asked to indicate their preferred product (“Which product do you prefer?”). If they had never tasted 1 or both of the items being compared, they were instructed to select the box “Never tasted one or both” and not indicate a preference.

Food neophobia is recurrent in the general population and in some population subgroups. It is detected by means of our questionnaire using an adapted version of the “Children Food Neophobia Scale” [30]. Specifically, respondents were asked to express their level of agreement or disagreement with 8 statements by selecting an answer between “No,” “Maybe,” and “Yes,” accompanied by pictures of a thumbs up or thumbs down.

Finally, we also included a section dedicated to proenvironmental behaviors, based on an adaptation of the “Children’s Self-Rated Pro-Environmental Behavior Scale” [31]. Respondents indicated their level of agreement or disagreement with statements related to performing daily activities in a sustainable way, selecting between “No,” “Maybe,” and “Yes,” with thumbs up or thumbs down images. The first item (“I carry out activities to protect the environment”) was optional.

Carers of individuals with DS were invited to complete the questionnaire designed for adults in Europe (see section Questionnaire for the General Population). Questionnaires from people with DS and their caregivers can be linked using a token generated by the respondents when they answer a set of common questions. When inconsistencies in the token matching are detected, the questionnaires are not linked. This approach allows for the exploration of any relationships between the habits, drivers, and barriers of people with DS and their caregivers.

### Statistical Analysis

Our primary data will undergo analysis using methods typically used to describe individual behaviors. This will involve multivariate analysis to delineate individual dietary patterns, purchasing and consumption behaviors, and discerning what respondents perceive as drivers and barriers to adopting healthier and more sustainable diets. Additionally, specific analysis will be conducted to provide answers to several research questions (Table 2). Examples of analyses that we are running on respondent data include, but are not limited to, regression analysis to identify variables that have the greatest association with healthy and sustainable food consumption; exploratory factor—and principal component—analyses to outline a map showing the distribution of participants according to perception, attitude, and sustainable behavior; and segmentation analysis to identify homogeneous segments within the dataset, thus facilitating the recognition of distinct groups, such as the delineation of “NEDS.” The research team will explore the use of confirmatory equation modeling or similar methods to examine the mediating role of selected drivers and barriers between food purchasing and consumption behaviors and the adoption of healthy and sustainable dietary patterns where supported by existing theoretical frameworks that explore these relationships. Among the analyses detailed in Table 2, survey weights will be applied only where weighted estimation is statistically appropriate and technically feasible (eg, descriptive statistics and regression models) and omitted where standard weighting procedures are not established (eg, Principal Component Analysis, Exploratory Factor Analysis, Confirmatory Factor Analysis, or Structural Equation Modeling).

**Table 2.** Primary research questions and corresponding analysis strategies, detailing key methodological components and subgroup analyses.

Matter	Research question	Primary outcome, independent variables, and geographical dimension	Analyses
Food behavior determinants	Is there a correlation between consumers’ self-reported food habits, priorities, beliefs, and individual socioeconomic, sociocultural, and contextual characteristics? Is there a geographical variation in the adoption of food diets consistent with planetary health principles across Europe? To what extent should the European “average consumer” paradigm be reconsidered to achieve effective empowerment in pursuing sustainability objectives, according to Directive (EU) 2024/825?	Primary outcome: consumers’ self-reported food habits Main exposures: priorities, beliefs, and individual socioeconomic, sociocultural, and contextual characteristics Geographical clustering: 5 food regions (Anglo-Saxon, Central European, Scandinavian, Southern European, and Eastern European)	Descriptive and inferential analyses (*P* value and ANOVA). Population segmentation, ANOVA and PCA^a^, linear regression models, documental and critical analyses of Directive (EU^b^) 2024/825 and its implementation measures, and descriptive and inferential analyses of survey data collected. Exploratory factor analysis and confirmatory analysis (CFA^c^/SEM^d^) will be performed to identify factors to use in the analyses.
Social stratification in food enjoyment	What are the relative within-country population-level shares of Europeans who view the foods they enjoy as (1) a barrier to their adoption of a healthier and more sustainable diet, (2) sometimes a barrier, and (3) not a barrier? Do responses linked to people viewing foods they enjoy as a barrier to adoption of a healthier and more sustainable diet vary within countries with regard to age, gender, education, income, and dietary patterns? Do dietary patterns vary by social class (income and education) in Europe in the ways that Bourdieusian’s studies demonstrate, that is, higher social classes prefer light, lean, bland foods, and lower social classes prefer fatty, meaty, salty foods?	Primary outcome: perception of “giving up foods that individuals like” as a barrier to healthier and sustainable diets Main exposures: age, gender, education, income, and dietary patterns Geographical clustering: national level	Mann-Whitney *U* tests to detect differences in response distributions among groups.
Vulnerable groups	Do vulnerable groups adopt diets that are consistent with planetary health diet principles? Which factors influence food purchasing and consumption among vulnerable groups? Which drivers and barriers are important in supporting vulnerable groups to adopt healthier and more sustainable diets? Are vulnerable groups likely to support policies oriented to healthier and more sustainable diets? Is there geographical variation in the dietary patterns of vulnerable groups across European countries?	Primary outcome: consumers’ self-reported food habits Main exposures: individual vulnerability (age, low income, low educational status, health status, family structure, and gender), factors perceived to influence food purchasing or consumption choices, factors facilitating or limiting the adoption of healthier and more sustainable diets, and intention to support policies addressing the barriers to adoption of a healthier and more sustainable food system. Geographical clustering: national level, 5 food regions	Descriptive statistics; *t* test or ANOVA for between-group comparisons; multivariate logistic, linear, ordinal, and regression; multilevel modeling to explore the relationship between the primary outcome and independent variables; and structural confirmatory equation modeling to confirm any mediator roles.
Food choice segments	What are the distinct subgroups of European adults based on factors impacting their food choices? Do sociodemographic characteristics predict subgroup membership? Do these profiles explain variability in eating behavior and primary food information sources?	Primary outcome: factors perceived to influence food purchasing or consumption choices. Main exposures: gender, education, perceived income, diet, and nutritional information sources. Geographical clustering: no	A 3-step latent profile analysis, Akaike Information Criterion, the Bayesian Information Criterion, the Lo‐Mendell‐Rubin likelihood ratio test, and entropy values.
Policy attitudes	What are the attitudes toward food policies that support the transition to healthier and more sustainable diets in European countries across different population subgroups? Is there a relationship between public attitude toward food policies supporting the transition to planetary health diet and existing diets of individuals? And with sociodemographic individual characteristics? And with factors influencing food purchasing?	Primary outcome: attitudes toward 10 food policies Main exposures: sociodemographics and health status, diets, and factors influencing food purchasing Geographical clustering: national	Descriptive statistics and ordinal regression models.

a PCA: Principal Component Analysis.

b EU: European Union.

c CFA: Confirmatory Factor Analysis.

d SEM: Structural Equation Modeling.

### Ethical Considerations

Ethical approval has been granted by Scuola Superiore Sant’Anna and Scuola Normale Superiore conjoined Ethics Committee: Resolution and following amendments n. 29/2023, 03/2024 and 41/2024. The results of the study will be disseminated through publications, reports, and conference presentations to target audiences across academia, nutrition and food practitioners, consumers, and policymakers. In light of the sensitive nature of the information collected in the sociodemographic section of the questionnaire, stringent measures have been implemented for data storage and analysis. To ensure security and compliance with General Data Protection Regulation regulations, all data have been stored and analyzed through remote access to a central server accessible only to named institutions in the approved ethics protocols and involved in the data analysis, designated as data processors, thus limiting the use of data externally. This approach significantly mitigates the risks associated with potential data breaches involving sensitive sociodemographic information. Access to the central server is strictly controlled and monitored, with robust encryption protocols in place to safeguard the confidentiality and integrity of the stored data. Additionally, measures have been implemented to anonymize and pseudonymize the data, further enhancing privacy protection.

## Results

### Study Timeline and Respondents

Data collection for the general population survey was conducted between February and June 2024, while recruitment for the survey targeting individuals with DS and their carers opened in March 2025 and closed in January 2026. In both cases, the recruitment and data collection periods coincided. Through the 27 European country survey, we collected 27,417 questionnaires across the European adults, 82 questionnaires from persons with DS, and 242 questionnaires from the caregivers of individuals with DS. Data analyses for the general population survey as well as those for individuals with DS and their carers are currently underway. The former analyses will be performed on 25,802 completed questionnaires that include the demographics necessary to weight the responses and compensate for deviations from the target sample strata sizes, ensuring that the final weighted sample approximates the population distribution. No missing data are present in this database. Table 3 describes the characteristics of the respondents. The remaining questionnaires were completed by participants who selected either “I prefer not to respond” or “I do not identify in one of the above [female or male] 2 genders” when asked about their gender identity. These data will be analyzed to understand food behaviors of the gender-diverse respondents’ subgroup.

**Table 3.** Sample characteristics (percentage) by age bands, sex, and educational attainment level, reported as unweighted (raw respondent sample) and weighted (adjusted for survey weights) percentages.

Demographics	Unweighted percentage	Weighted percentage
Age bands (years)		
18-34	23.95	22.25
35-44	18.47	16.73
45-54	19.25	17.89
55-74	32.29	30.90
75 and older	6.04	12.23
Sex		
Female	49.99	51.66
Male	50.01	48.34
Education		
0‐2^a^	16.20	22.58
3‐4^a^	48.22	44.43
5‐8^a^	35.58	32.98

a The International Standard Classification of Education classification of education comprises 9 levels (0-8). For the purposes of this table, these levels were aggregated into the broader categories commonly adopted for the ordinal reporting of educational attainment.

## Discussion

### Principal Contributions

The ambition of this study is to map the food behaviors of Europeans, assess their consistency with the planetary health diet principles, and obtain insights on which factors influence individual food choices. To the best of our knowledge, unlike others [32,33] this is one of the few studies [34] that simultaneously examines food consumption patterns, purchasing behaviors, drivers and barriers to healthy and sustainable diets, and public support for food policies in a large, geographically diverse sample of European adults. This multidimensionality enables a form of analysis that was not possible in previous European dietary surveys: rather than studying food consumption in isolation [35,36], this study allows researchers and policymakers to understand whether and how dietary patterns and their determinants are linked across the subgroups of the population individuals using the same measures.

To also provide consistent results at the country level, we designed a cross-sectional survey collecting data from 1000 respondents per country using a stratified sample design, with the aim of reflecting the real-world demographic composition of each national population. We recruited respondents through market research panels that are consistent in ensuring participation across all sample strata.

Importantly, this depth of coverage also allows us to explore the food habits and attitudes of vulnerable groups, including older adult individuals, people with lower income or educational attainment, those living with chronic health conditions or with disabilities (specifically individuals with DS), households with minors, women, and gender-diverse people. This makes it possible to compare the relative vulnerability of different vulnerable subgroups on a common scale, rather than drawing on evidence from separate studies that are methodologically incomparable.

Regarding the tools we used to map the food habits of Europeans’ food, a valuable methodological contribution of this work is the development of the ELFI index [19], which converts frequency-of-consumption data into a measure of conformity to planetary health diet principles. This was a deliberate choice: we are aware that frequency measures cannot quantify the individual adherence to the EAT-Lancet recommendations, but at the same time, large-scale and multidimensional cross-national surveys cannot realistically rely on quantitative intake measurements, and frequency-based approaches have been shown to perform well in this context in comparison with other intake-based measurements [19,37]. Indeed, the ELFI index makes it possible to benchmark European dietary behaviors against the planetary health diet principles in a way that has been statistically validated [19].

Regarding the expected findings, they may describe to what extent European dietary habits are aligned with planetary health diet principles and whether there are different habits depending on specific foods or nutrients, suggesting potential (positive or negative) impacts on the population and planetary health. The findings also have the potential to reveal potential misalignments between people’s reported diets, their perception of the health and sustainability of these diets [38], and the achievement of health goals for individuals and the planet, pointing out any room for action at all levels of the food system.

The findings on purchasing behaviors, drivers, and barriers to healthier and more sustainable diets may reveal a complex picture and contribute to understanding why Europeans eat the way they do and, for example, whether healthier and more sustainable options are always perceived as more expensive and less accessible than unhealthy and unsustainable options [39]. Variation on different attributes (eg, geographical location and for different population subgroups) will also be explored.

The perceived drivers and barriers data may add important nuances and create new knowledge about the co-occurrence of multiple factors dealing with individual, social, and structural facilitators and barriers. Furthermore, the results of both the dietary habits and the drivers and barriers will be explored based on different attributes (eg, geographical location and different population subgroups) to understand variation that could then inform context-dependent and context-independent policy and practice measures to support adherence to planetary health diet principles.

On the policy side, the results may point out whether and to what extent the European population is supportive of food policy interventions and whether this support is stronger or weaker depending on the type of action proposed. These findings could have direct relevance for policymakers working within the framework of the EU “Vision for Agriculture and Food” Strategy and the “Safe Hearts Plan” and show whether there is support or resistance to food policy reform at the level of different European countries as well as the EU more broadly. Furthermore, these findings may serve as a starting point to understand the feasibility of implementing these policies politically and administratively at both the EU and member state levels. These findings also serve as inputs for other domains of the FEAST project (Figure 3), including our simulations and modeling work package that aims to elucidate the health, social, environmental, and economic benefits and trade-offs of different policy options (eg, 0% VAT on fruits and vegetables, taxes on red meat, and control of social media interventions), as well as our work package focused on policy dialogues that aims to understand the barriers and facilitators to policymakers designing and implementing policies that support Europe’s food systems to become healthier and more sustainable at macro-, meso-, and microlevels.

**Figure 3.**
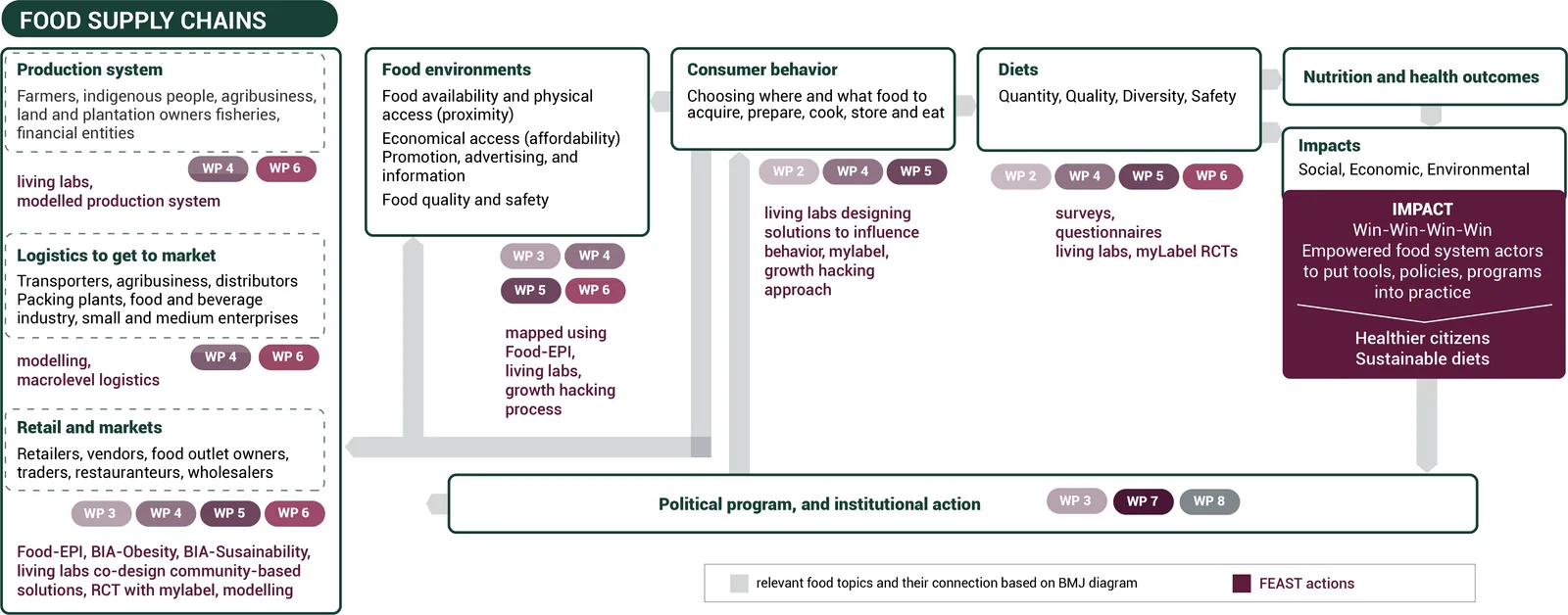
Domains of the FEAST project informed by the findings of this study and supporting meaningful impact on food system transition (adapted with permission from FEAST). BIA: Business Impact Assessment; EPI: Environment Policy Index; FEAST: Food Systems That Support Transitions to Healthy and Sustainable Diets; RCT: randomized controlled trial.

Finally, further analyses may contribute to investigating the complex interrelationships between factors linked to the action of different levels of the food system and identify (warranted and unwarranted) variation in access and consumption among vulnerable groups.

### Limitations

There are several limitations that need to be acknowledged about our study design. The use of panels for a study such as ours introduces potential selection bias that may affect the representativeness of certain subgroups [40]. It is not feasible to account for all demographic attributes (eg, for our panel provider, they did not have information about the income levels of panel members), but for our study, we attempted to account for this by setting quotas for recruitment based on Eurostat data on different demographic attributes. Furthermore, for other attributes, we included specific questions (eg, income level) in order to gather information that would allow us to first understand the representation of these groups in our sample as well as to conduct subgroup analyses on these different population subgroups, many of which represent different vulnerabilities.

Another set of limitations is linked to questionnaire items. Food groups and nutrients monitored do not include sodium, saturated fatty acids, or fiber-rich products, producing a gap in the comprehensive monitoring of healthy and sustainable diets. Furthermore, self-reported data on behaviors, intentions, barriers, and facilitators linked to particular behaviors and preferences are at risk of susceptibilities to recall bias, social projection bias, and social desirability bias [41,42]. These risks of bias are common in studies such as ours, which was done at such a large scale, where objective measures are not available or feasible to collect. For our study, the large-scale and cross-sectional design can provide some protection against these biases, particularly for the conclusions that are very robust and obvious (eg, the preference for 0% VAT on fruits and vegetables), but we acknowledge that caution and nuance are necessary for analyses linked to findings that are not so obvious.

### Conclusions

This study, with over 27,000 respondents across 27 European countries, goes beyond what most existing research in this area has achieved (limited number of countries, few dimensions of dietary behavior, or specific population subgroups, requiring harmonization processes to bring together data from multiple sources and build up a comprehensive overview). This multidimensional exploration can provide important insights on the structural, social, and individual barriers that are associated with the adoption of healthier and more sustainable diets. Understanding the potential public support for removing formal and de facto barriers through new policies may complement individual profiles, potentially capturing possible preferences for, and engagement with, a healthier and more sustainable food system that could go beyond individual behavioral effort and make the healthiest and most sustainable option the most desirable, easiest, accessible, and affordable one.

These forthcoming results will contribute to the broad debate on the promotion of healthier and more sustainable food behaviors and offer a rich and comprehensive picture of European food habits, the factors shaping food choices, and the areas where the greatest opportunities for interventions lie at the micro-, meso-, and macrolevels of the food system. The new findings will help redesign the food system and actively support the transition toward a system that prioritizes planetary health and in which all key stakeholders in the food system (people, the environment, the public sector, and small, medium, and large private sector actors) contribute through a process of value exchange. These results will also stimulate further research by scholars aimed at guiding the food system toward the adoption of healthy and sustainable food behaviors.

## Supplementary material

10.2196/91985 Multimedia Appendix 1 Questionnaires.

## Abbreviations

DS: Down syndrome
ELFI: EAT-Lancet Consumption Frequency Index
EU: European Union
FEAST: Food Systems that Support Transitions to Healthy and Sustainable Diets
ISCED: International Standard Classification of Education
NEDS: Natural European Dietary Segments
VAT: value-added tax
