# Supplementary material for: Exploring Perceived Barriers and Facilitators to Healthier and More Sustainable Dietary Behavior in 27 European Countries: Protocol for a Cross-Sectional Survey
Source: JMIR Res Protoc. 2026 Sep 30;15:e91985. doi: 10.2196/91985 (PMC13626401; doi:10.2196/91985)
Supplement: Supplementary file 1 [file resprot-v15-e91985-s001.pdf]

FEAST Questionnaire: **"Explore adherence to and factors influencing healthy and sustainable dietary choices across Europe"**

A) *DIETARY PATTERN (14 questions)*

Q1 Select your frequency of consumption for each food group in the last month

Q1.01 **Whole grains** (Rice, wheat, corn, bread and other whole grain products in the form of breakfast cereals, biscuits, tortillas and other sources) *(Not including refined versions of these products, such as white rice and white bread)* and **Pseudograins** (Amaranth, Buckwheat, Quinoa)

1. Never
2. One to three times a month
3. Once per week
4. From 2 to 3 times a week
5. From 4 to 6 times a week
6. Daily (one time a day)
7. Daily (2 or more times a day)

Q1.02 **Tubers or Starches** (Potatoes, cassava and their derivatives, such as potato flour and instant mashed potatoes)

1. Never
2. One to three times a month
3. Once per week
4. From 2 to 3 times a week
5. From 4 to 6 times a week
6. Daily (one time a day)
7. Daily (2 or more times a day)

Q1.03 **Vegetables** (all vegetables, excluding legumes and starchy vegetables)

1. Never
2. One to three times a month
3. Once per week
4. From 2 to 3 times a week
5. From 4 to 6 times a week
6. Daily (one time a day)
7. Daily (2 or more times a day)

Q1.04 **Fruits** (All fruits and berries, excluding fruit juices)

1. Never
2. One to three times a month
3. Once per week
4. From 2 to 3 times a week
5. From 4 to 6 times a week

6. Daily (one time a day)
7. Daily (2 or more times a day)

Q1.05 **Dairy Foods** (milk, yogurt, cheese, from cow, goat or buffalo) *(excluding butter and cream)*

1. Never
2. One to three times a month
3. Once per week
4. From 2 to 3 times a week
5. From 4 to 6 times a week
6. Daily (one time a day)
7. Daily (2 or more times a day)

Q1.06 **Beef, lamb, goat, pork, and red meat in its processed form** (e.g., sausages, ham, bologna, dried meat, etc.)

1. Never
2. One to three times a month
3. Once per week
4. From 2 to 3 times a week
5. From 4 to 6 times a week
6. Daily (one time a day)
7. Daily (2 or more times a day)

Q1.07 **Chicken and other poultry** (e.g. duck, geese, turkey)

1. Never
2. One to three times a month
3. Once per week
4. From 2 to 3 times a week
5. From 4 to 6 times a week
6. Daily (one time a day)
7. Daily (2 or more times a day)

Q1.08 **Eggs** (from chicken and other poultry)

1. Never
2. One to three times a month
3. Once per week
4. From 2 to 3 times a week
5. From 4 to 6 times a week
6. Daily (one time a day)
7. Daily (2 or more times a day)

Q1.09 **Fish and shellfish** (e.g., mussels, shrimp, octopus)

1. Never
2. One to three times a month
3. Once per week
4. From 2 to 3 times a week
5. From 4 to 6 times a week
6. Daily (one time a day)
7. Daily (2 or more times a day)

Q1.10 **Legumes** (e.g. dried or canned beans, lentils, chickpeas, peas, soy, tofu, soy products)

1. Never
2. One to three times a month
3. Once per week
4. From 2 to 3 times a week
5. From 4 to 6 times a week
6. Daily (one time a day)

7. Daily (2 or more times a day)

Q1.11 **Nuts** (e.g., pistachios, walnuts, hazelnuts, almonds, peanuts, nut mixes) *(excluding sweetened and salted nuts*)

1. Never
2. One to three times a month
3. Once per week
4. From 2 to 3 times a week
5. From 4 to 6 times a week
6. Daily (one time a day)
7. Daily (2 or more times a day)

Q1.12 **All plant oils** (e.g. olive oil, sunflower oil, soybean oil, etc.) and **plant margarines**

1. Never
2. One to three times a month
3. Once per week
4. From 2 to 3 times a week
5. From 4 to 6 times a week
6. Daily (one time a day)
7. Daily (2 or more times a day)

Q1.13 **Animal fats and other saturated fats** (butter, tallow, lard, coconut oil, cream)

1. Never
2. One to three times a month
3. Once per week
4. From 2 to 3 times a week
5. From 4 to 6 times a week
6. Daily (one time a day)
7. Daily (2 or more times a day)

Q1.14 **Added sugars** (all sugars – white, brown, honey, agave syrup, maple syrup), **sweets/desserts, fruit-juices and sugar-sweetened beverages (e.g. soda)**

1. Never
2. One to three times a month
3. Once per week
4. From 2 to 3 times a week
5. From 4 to 6 times a week
6. Daily (one time a day)
7. Daily (2 or more times a day)

B) PURCHASING AND CONSUMPTION (21 questions)

Q2) In an average week, how often are you the person in charge of household grocery purchases?

1. Never
2. Rarely
3. Sometimes
4. Often
5. Always

Q3) In an average week, select place(s) where most household food shopping comes from *(please check all that apply)*

1. Independent stores, Butchers, Grocers

2. One supermarket
3. Two or more supermarkets
4. Local markets (Including farmer’s markets)
5. Direct from a farmer or community supported agriculture
6. Online grocery delivery
7. Food banks/community larders

Q4) In an average week, how often are you the person in charge of cooking/preparing meals in the household?
1. Never
2. Rarely
3. Sometimes
4. Often
5. Always

Q5) In an average week, where do most of your meals come from? *(please check all that apply) (Multiple choice)*
1. Prepared at home
2. Ready made meals from shops/supermarkets
3. Restaurants
4. Canteen
5. Takeaway
6. Meal delivery
7. Food banks/community larders

Q6) How often do you consider the following elements when purchasing/consuming food?

Q6.01 Good quality for its price
1. Never
2. Rarely
3. Sometime
4. Often
5. Always

Q6.02 Clear ingredients list
1. Never
2. Rarely
3. Sometime
4. Often
5. Always

Q6.03 Nutritional value of the product
1. Never
2. Rarely
3. Sometime
4. Often
5. Always

Q6.04 Packaging appearance (Color, use of pictures, overall design)
1. Never
2. Rarely
3. Sometime
4. Often
5. Always

Q6.05 Packaging size
1. Never
2. Rarely
3. Sometime
4. Often
5. Always

Q6.06 Environmentally friendly packaging
1. Never
2. Rarely
3. Sometime
4. Often
5. Always

Q6.07 Clear information about the country of origin
1. Never
2. Rarely
3. Sometime
4. Often
5. Always

Q6.08 Ease of preparation
1. Never
2. Rarely
3. Sometime
4. Often
5. Always

Q6.09 Availability where I usually shop for food
1. Never
2. Rarely
3. Sometime
4. Often
5. Always

Q6.10 What I'm typically used to eating
1. Never
2. Rarely
3. Sometime
4. Often
5. Always

Q6.11 Religious beliefs
1. Never
2. Rarely
3. Sometime
4. Often
5. Always

Q6.12 Seasonality of the food
1. Never
2. Rarely
3. Sometime
4. Often
5. Always

Q6.13 Impact on the environment (greenhouse gas emissions, biodiversity loss, deforestation, water use, etc.)
1. Never
2. Rarely
3. Sometime
4. Often
5. Always

Q6.14 Certified for ethical and/or sustainable production (fairtrade, MSC, rain forest alliance certified)
1. Never
2. Rarely
3. Sometime
4. Often
5. Always

Q6.15 Eating habits of others around me
1. Never
2. Rarely

3. Sometime
4. Often
5. Always

Q6.16 Food produced locally

1. Never
2. Rarely
3. Sometime
4. Often
5. Always

Q6.17 Overall liking of the product (appearance, odour, taste, flavour, texture)

1. Never
2. Rarely
3. Sometime
4. Often
5. Always

C) SUSTAINABILITY (2 questions)

Q7) How often would you say that:

Q7.01 *Personally, you eat a healthy and sustainable diet?*

1. Never
2. From time to time
3. Most of the time
999. I Don't know

Q7.02 *Others in your social circle eat a healthy and sustainable diet?*

1. Never
2. From time to time
3. Most of the time
999. I Don't know

D) NUTRITIONAL INFORMATION (1 question)

Q*8) Select up to three of your main sources of nutritional information*

1. Family
2. Friends/Peers/Neighbours
3. Traditional media (e.g. TV, Radio, Newspapers, Books, Magazines, etc.)
4. Online Resources (e.g. Social Media, Google searches, Youtube, etc.)
5. School/Work colleagues/Everyday places
6. Healthcare professionals (e.g. nutritionists, dieticians, doctors, nurses, etc.)
7. Consumer-related (e.g. supermarkets, food labels, food packaging, etc.)
8. Government/Health authorities (e.g. National dietary guidelines, international dietary recommendations)
9. Other: Please specify_____________

E) FACILITATORS (8 questions)

Q9*) *To what extent do these elements support you to eat a healthy and sustainable diet?

Q9.01 My knowledge about healthy and sustainable diets

1. Not at all
2.
3.
4.
5. Completely

Q9.02 Availability of practical supportive tools (e.g., Menu guides, Apps, scoring systems…)

1. Not at all
2.
3.
4.
5. Completely

Q9.03 Availability of healthy and sustainable food choices where I usually eat or shop for food

1. Not at all
2.
3.
4.
5. Completely

Q9.04 Expectations for me to be healthy by people who are important to me

1. Not at all
2.
3.
4.
5. Completely

Q9.05 External pressures to eat a healthy and sustainable diet (social media, policy, social campaigns, healthy eating guidelines..)

1. Not at all
2.
3.
4.
5. Completely

Q9.06 Healthy and sustainable eating habits/behaviors of people I share my meals with

1. Not at all
2.
3.
4.
5. Completely

Q9.07 Personal goal to improve my health

1. Not at all
2.
3.

4.
5. Completely

Q9.08 Availability of information on the sustainable and ethical production of the food item

1. Not at all
2.
3.
4.
5. Completely

F) BARRIERS (10 questions)

Q10*) *To what extent do these elements limit you to eat a healthy and sustainable diet?

Q10.01 My irregular working hours

1. Not at all
2.
3.
4.
5. Completely

Q10.02 My busy lifestyle

1. Not at all
2.
3.
4.
5. Completely

Q10.03 Giving up foods that I like

1. Not at all
2.
3.
4.
5. Completely

Q10.04 Having to eat strange or unusual foods

1. Not at all
2.
3.
4.
5. Completely

Q10.05 Unaffordability of healthy and sustainable food choices

1. Not at all
2.
3.
4.
5. Completely

Q10.06 My difficulty in preparing healthy and sustainable meals

1. Not at all
2.

3.
4.
5. Completely

Q10.07 My lack of willpower

1. Not at all
2.
3.
4.
5. Completely

Q10.08 My lack of knowledge about healthy and sustainable eating

1. Not at all
2.
3.
4.
5. Completely

Q10.09 Unhealthy and unsustainable eating habits/behaviours of people I share my meals with

1. Not at all
2.
3.
4.
5. Completely

Q10.10 Unavailability of healthy and sustainable food choices close to where I live/work/spend most of my time

1. Not at all
2.
3.
4.
5. Completely

G) POLICY QUESTIONS (10 questions)

We are interested in your opinion about food policies. For each statement, please indicate whether you would support or oppose the policy.

Q11*) *Would you support or oppose a government policy that would require…

Q11**.**01 Increasing taxes for products with higher added sugar

1. Oppose
2. Neutral
3. Support

888. I prefer not to respond
999. I don't know

Q11.02 0% VAT on fruits and vegetables

1. Oppose
2. Neutral
3. Support

888. I prefer not to respond
999. I don't know

Q11.03 *Ban* on price discounts for unhealthy food and beverages (e.g., 30% off, or 'buy-one-get-one-free')

1. Oppose
2. Neutral
3. Support
888. I prefer not to respond
999. I don't know

Q11.04 *Ban* of marketing of unhealthy food and beverages (e.g., sugary drinks, crisps, chocolate) on all media including social media/ the internet/TV/radio/ newspapers/ magazines

1. Oppose
2. Neutral
3. Support
888. I prefer not to respond
999. I don't know

Q11.05 *Ban* of unhealthy food and beverages (e.g., sugary drinks, crisps, chocolate) at supermarket checkouts

1. Oppose
2. Neutral
3. Support
888. I prefer not to respond
999. I don't know

Q11.06 *Ban* of marketing of unhealthy food and beverages (e.g., sugary drinks, crisps, chocolate) to children through the use of cartoon characters and other appealing elements on packaging

1. Oppose
2. Neutral
3. Support
888. I prefer not to respond
999. I don't know

Q11.07 *Ban* of outdoor advertisements (e.g., at bus stops and billboards) for unhealthy food and beverages (e.g., sugary drinks, crisps, chocolate)

1. Oppose
2. Neutral
3. Support
888. I prefer not to respond
999. I don't know

Q11.08 Ban on sales of energy drinks to people under 18 years of age

1. Oppose
2. Neutral
3. Support
888. I prefer not to respond
999. I don't know

Q11.09 Taxes on red meat production

1. Oppose
2. Neutral
3. Support
888. I prefer not to respond
999. I don't know

Q11.10 Labels about environmental sustainability (e.g., carbon footprint, harm on wildlife and biodiversity) on packaged foods

888. I prefer not to respond
999. I don't know

1. Oppose
2. Neutral
3. Support

H) WHO IS RESPONDING (22 questions)

Q12) Age: *Insert age in completed years* ___________________

Q13) Level of education:

1. Early childhood education *(hover over "?" to show national equivalencies)*
2. Primary education *(hover over "?" to show national equivalencies)*
3. Lower secondary education *(hover over "?" to show national equivalencies)*
4. Upper secondary education *(hover over "?" to show national equivalencies)*
5. Post-secondary non-tertiary education *(hover over "?" to show national equivalencies)*
6. Short-cycle tertiary education *(hover over "?" to show national equivalencies)*
7. Bachelor's or equivalent *(hover over "?" to show national equivalencies)*
8. Master's or equivalent *(hover over "?" to show national equivalencies)*
9. Doctorate or equivalent *(hover over "?" to show national equivalencies)*

Q14) Gender identity

1. Female
2. Male
3. I do not identify in one of the above two genders

888. I prefer not to respond

Q15) How would you describe your principal occupational status?

1. Unemployed
2. Unable to work due to long standing health problems
3. Retired
4. Working part-time
5. Working full-time

Q16) What is your household net yearly income? (€)

1. *up to 2999*
2. *3000-3999*
3. *4000-4999*
4. *5000-5999*
5. *6000-6999*
6. *7000-8499*
7. *8500-10999*
8. *11000-12499*
9. *12500-14999*
10. *15000-17499*
11. *17500-19999*
12. *20000-22499*

13. *22500-24999*
14. *25000-27499*
15. *27500-29999*
16. *30000-34999*
17. *35000-39999*
18. *40000-44999*
19. *45000-49999*
20. *50000-54999*
21. *55000-59999*
22. *60000-69999*
23. *70000-79999*
24. *80000-89999*
25. *90000-99999*
26. *Above 100000*

Q17) Do you feel your household income is sufficient to meet your living expenses (e.g. food, housing, energy costs, heating/cooling, travel, etc.)?

1. No
2. Sometimes
3. Yes

Q18) How many people live in your household (including you): *Insert total number of individuals in household*_________________

Q19) How many individuals living in your household are under 18 years old? *Insert number of individuals living in your household under 18 years old*__________

Q20) How many individuals living in your household are above 65 years old (including you)? *Insert number of individuals living in your household over 65 years old*________________

Q21) Country of birth

1. Afghanistan (AF)
   …..
225. Zimbabwe (ZW)

Q22) Country of birth of parent 1 (*Optional)

1. Afghanistan (AF)
   …..
225. Zimbabwe (ZW)

Q23) Country of birth of parent 2 (*Optional)

1. Afghanistan (AF)
   …..
225. Zimbabwe (ZW)

Q24) Country you spend most of your time in

1. Afghanistan (AF)
   …..
225. Zimbabwe (ZW)

Q25) How much time have you spent in the above country? *Insert time in completed years*_________________

Q26) Would you describe the place where you live as...

1. A city
2. The suburbs or outskirts of a big city
3. A small city or town
4. A country village
5. A farm or home in the country

888. I prefer not to respond

Q27) ZIP code of your place of residence:

*Insert ZIP CODE o*f place of residence________________________

888. I prefer not to respond

Q28) Would you describe the place where you work/study/spend most of your time outside of the place you live as…

1. A city
2. The suburbs or outskirts of a big city
3. A small city or town
4. A country village
5. A farm or home in the country
6. I do not have a fixed job/study/usual place where I spend most of my time outside of home

888. I prefer not to respond

Q29) ZIP CODE of your place of work or frequent/usual activity*:*

Insert ZIP CODE of place of work ______________________

888. I prefer not to respond

Q30) Do you have any pre-existing health conditions (please check all that apply):

1. I don't have pre-existing health conditions
2. Mental Health Illness (Depression/Anxiety; severe mental illness, e.g. schizophrenia, bipolar disorder)
3. Metabolic disorder (Type 1 Diabetes, Type 2 Diabetes, Obesity)
4. Cardiovascular disease (high blood pressure, high cholesterol, cardiovascular disease)
5. Respiratory disorder (e.g. asthma, COPD)
6. Cancer
7. Gastrointestinal condition (e.g. irritable bowel syndrome)
8. Muskuloskeletal disorders (e.g. osteoporosis, arthritis, sarcopenia, etc.)
9. Other (Please Specify)__________________

888. I prefer not to respond

Q31) Your Height*: insert height*_______________ *(cm)*

Q32) Your Weight*: insert weight*______________*(kg)*

FEAST questionnaire: **Food Choices among Down Syndrome Individuals.**

1.Let's get to know you

1.1 Gender:

a. Male b. Female c. Other

1.2 Age.

Please write your answer here: |__|

1.3 How many meals do you consume in the following locations:

	Never	Some of my meals	Most of my meals
**1.3.1 At home**			
**1.3.2 Canteen**			
**1.3.3 Other locations (e.g. Restaurant/Fast food)**			

2. Food Frequency Questionnaire

How often have you eaten these foods in the last month?		
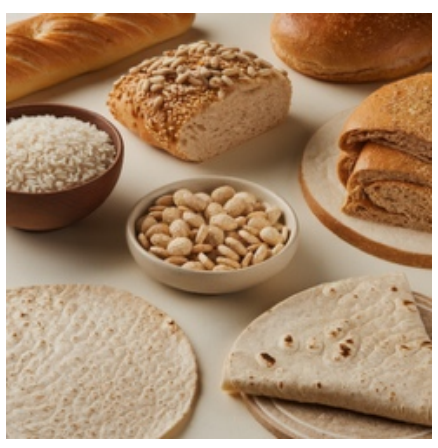	**2.1 Brown rice, whole wheat, wheat, corn, whole-wheat bread, whole-wheat breakfast cereals, whole wheat biscuits, whole-wheat/ corn tortillas, quinoa.**	• Never<br>• Once to twice a month<br>• Once per week<br>• From 2 to 3 times a week<br>• From 4 to 6 times a week<br>• Daily (one time a day)<br>• Daily (2 or more times a day)
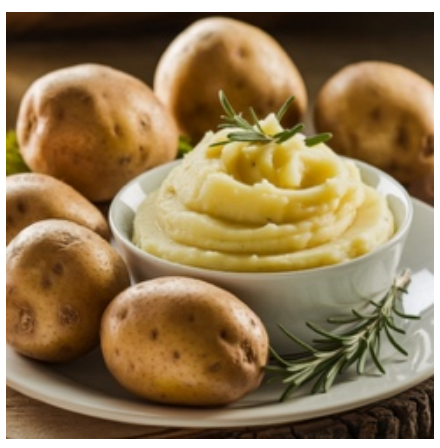	**2.2 Potatoes and mashed potatoes.**	• Never<br>• Once to twice a month<br>• Once per week<br>• From 2 to 3 times a week<br>• From 4 to 6 times a week<br>• Daily (one time a day)<br>• Daily (2 or more times a day)

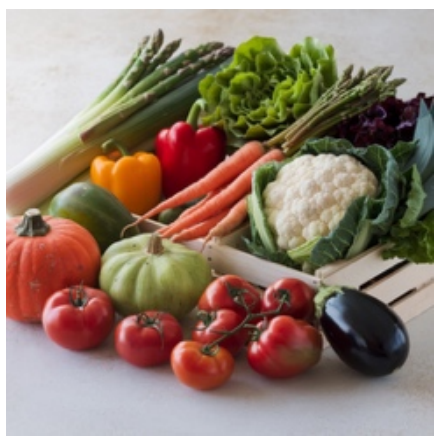	**2.3 Vegetables**	• Never<br>• Once to twice a month<br>• Once per week<br>• From 2 to 3 times a week<br>• From 4 to 6 times a week<br>• Daily (one time a day)<br>• Daily (2 or more times a day)
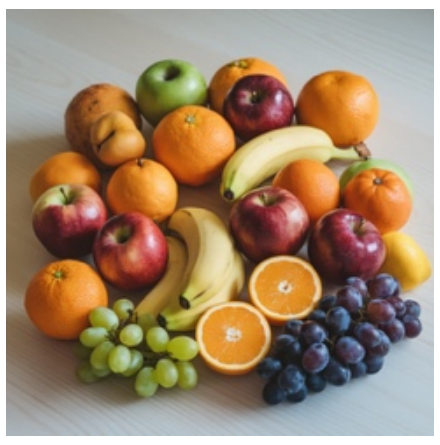	**2.4 Fruit**	• Never<br>• Once to twice a month<br>• Once per week<br>• From 2 to 3 times a week<br>• From 4 to 6 times a week<br>• Daily (one time a day)<br>• Daily (2 or more times a day)
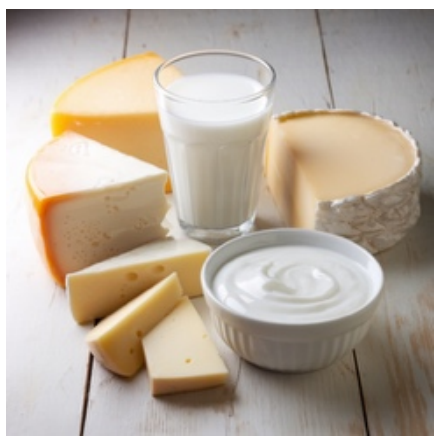	**2.5 Dairy products (milk, cheese, yoghurt)**	• Never<br>• Once to twice a month<br>• Once per week<br>• From 2 to 3 times a week<br>• From 4 to 6 times a week<br>• Daily (one time a day)<br>• Daily (2 or more times a day)
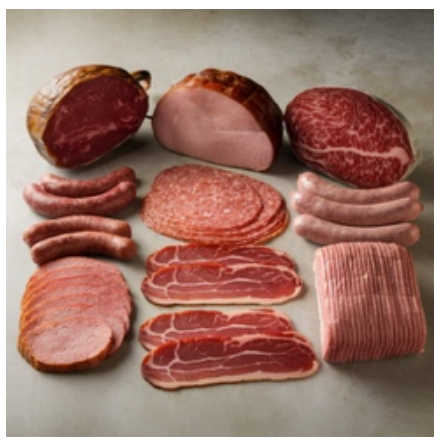	**2.6 Red meat and cold meats, ham, salami, sausages, bacon, mince**	• Never<br>• Once to twice a month<br>• Once per week<br>• From 2 to 3 times a week<br>• From 4 to 6 times a week<br>• Daily (one time a day)<br>• Daily (2 or more times a day)
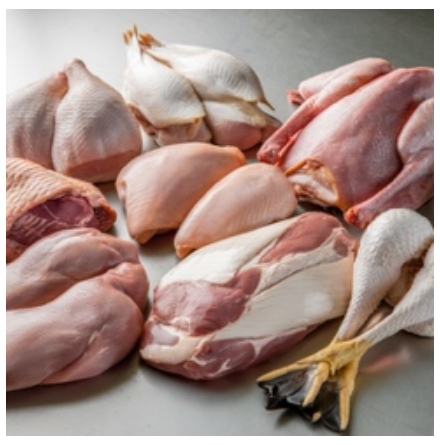	**2.7 Chicken, duck, goose, turkey**	• Never<br>• Once to twice a month<br>• Once per week<br>• From 2 to 3 times a week<br>• From 4 to 6 times a week<br>• Daily (one time a day)<br>• Daily (2 or more times a day)
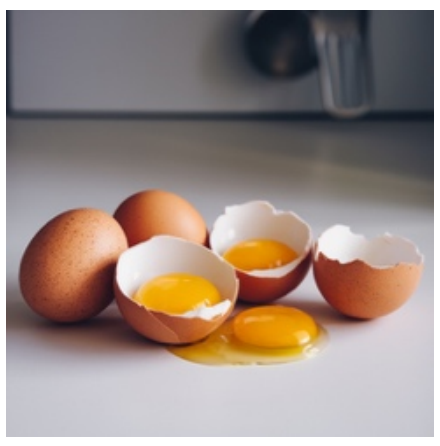	**2.8 Eggs**	• Never<br>• Once to twice a month<br>• Once per week<br>• From 2 to 3 times a week<br>• From 4 to 6 times a week<br>• Daily (one time a day)<br>• Daily (2 or more times a day)
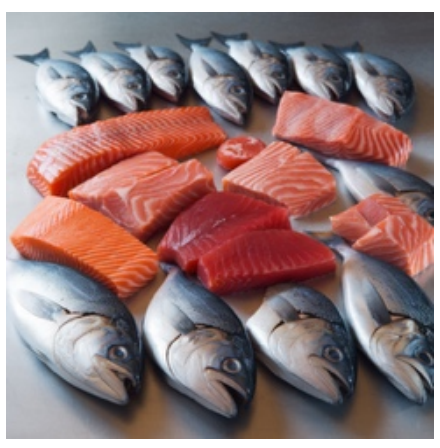	**2.9 Fish**	• Never<br>• Once to twice a month<br>• Once per week<br>• From 2 to 3 times a week<br>• From 4 to 6 times a week<br>• Daily (one time a day)<br>• Daily (2 or more times a day)

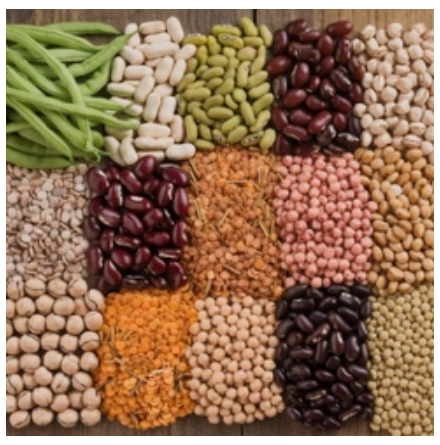	**2.10 Beans (Greenbeans, kidney, lentils, beans chickpeas)**	• Never<br>• Once to twice a month<br>• Once per week<br>• From 2 to 3 times a week<br>• From 4 to 6 times a week<br>• Daily (one time a day)<br>• Daily (2 or more times a day)
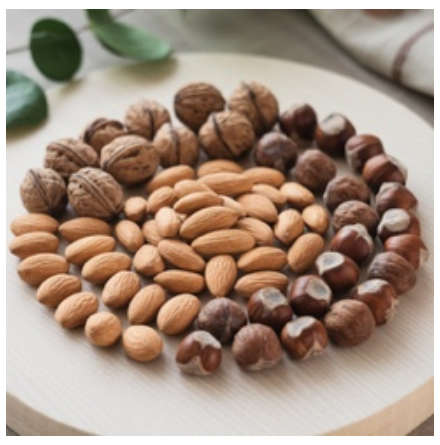	**2.11 Nuts**	• Never<br>• Once to twice a month<br>• Once per week<br>• From 2 to 3 times a week<br>• From 4 to 6 times a week<br>• Daily (one time a day)<br>• Daily (2 or more times a day)
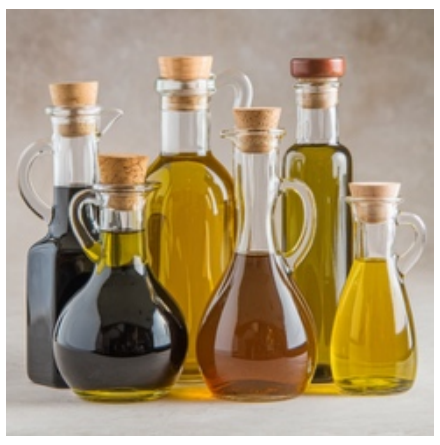	**2.12 Virgin olive oil, olive oil, sunflower oil, soybean oil, almond oil**	• Never<br>• Once to twice a month<br>• Once per week<br>• From 2 to 3 times a week<br>• From 4 to 6 times a week<br>• Daily (one time a day)<br>• Daily (2 or more times a day)
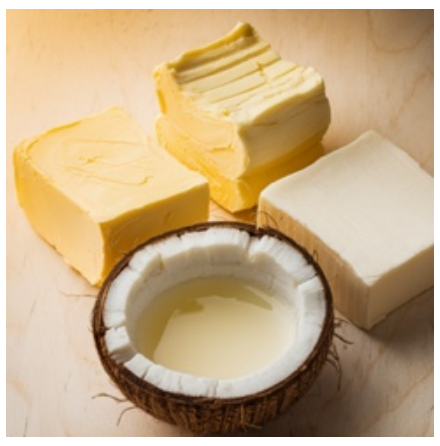	**2.13 Butter, coconut oil, lard**	• Never<br>• Once to twice a month<br>• Once per week<br>• From 2 to 3 times a week<br>• From 4 to 6 times a week<br>• Daily (one time a day)<br>• Daily (2 or more times a day)
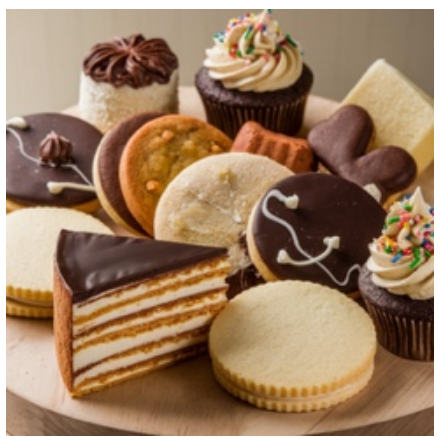	**2.14 Bakery and sweets**	• Never<br>• Once to twice a month<br>• Once per week<br>• From 2 to 3 times a week<br>• From 4 to 6 times a week<br>• Daily (one time a day)<br>• Daily (2 or more times a day)
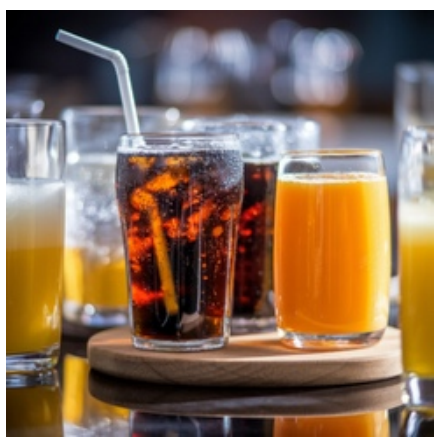	**2.15 Carbonated beverages (e.g. fizzy juice)**	• Never<br>• Once to twice a month<br>• Once per week<br>• From 2 to 3 times a week<br>• From 4 to 6 times a week<br>• Daily (one time a day)<br>• Daily (2 or more times a day)
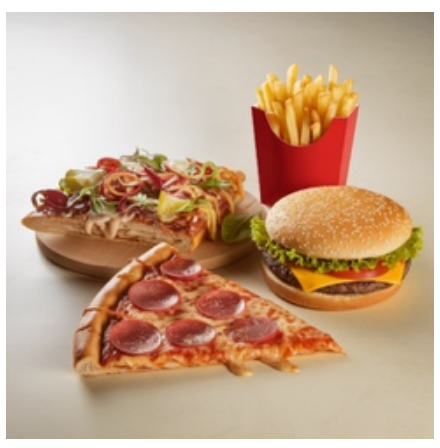	**2.16 Fast food (Pizza, hamburger, French fries)**	• Never<br>• Once to twice a month<br>• Once per week<br>• From 2 to 3 times a week<br>• From 4 to 6 times a week<br>• Daily (one time a day)<br>• Daily (2 or more times a day)

3.What do you look for in food?

**It is important to me that the food I eat...**

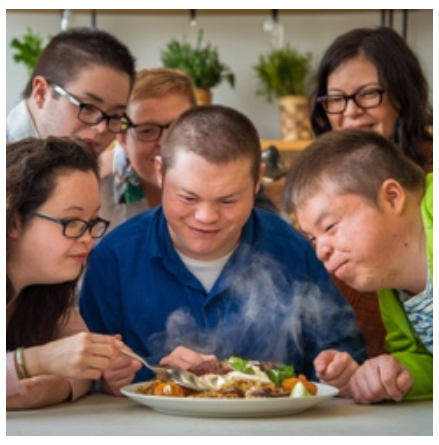	**3.1 Smells nice**	Not important	Maybe	Important
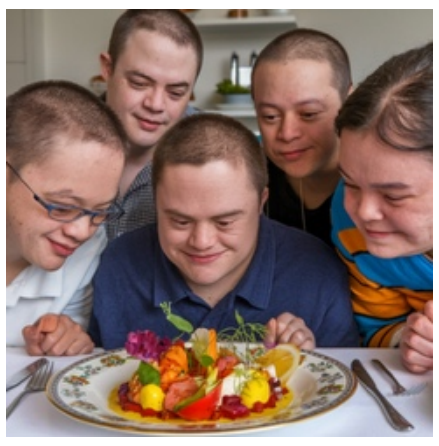	**3.2 Looks nice**	Not important	Maybe	Important
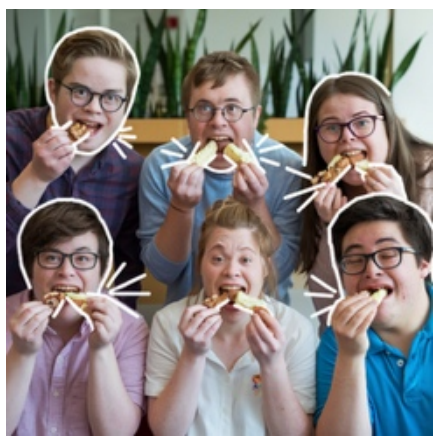	**3.3 Has a nice texture**	Not important	Maybe	Important
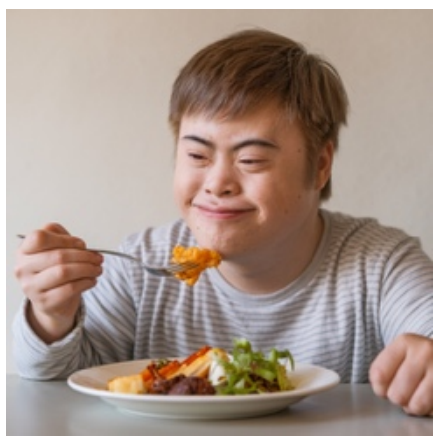	**3.4 Tastes good**	Not important	Maybe	Important

4. Habits

**It is important to me that the food I eat...**				
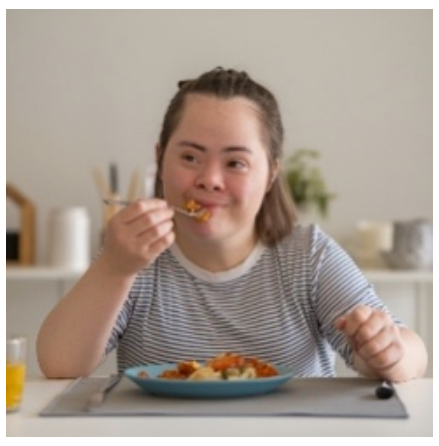	**4,1 I eat small portions of food to help control my weight.**	No	Maybe	Yes
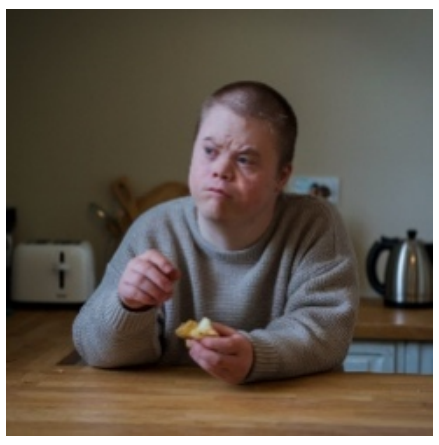	**4.2 I eat when I feel worried.**	No	Maybe	Yes
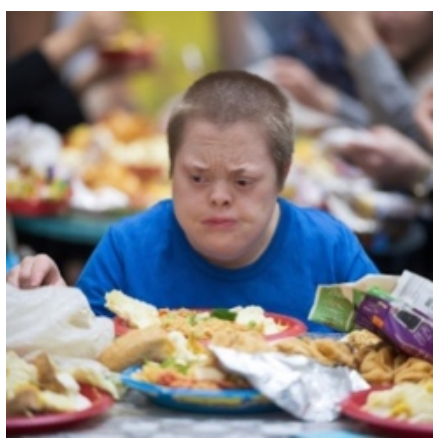	**4.3 Sometimes when I start eating, it seems I can't stop.**	No	Maybe	Yes

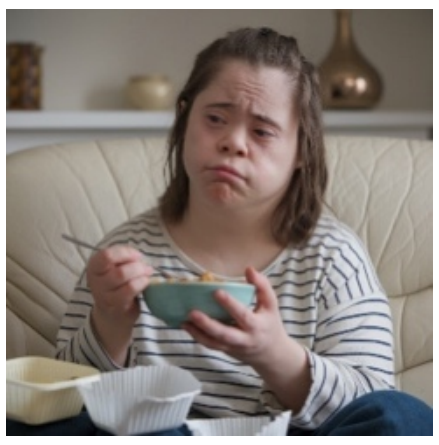	**4.4 When I am sad, I usually eat too much.**	No	Maybe	Yes
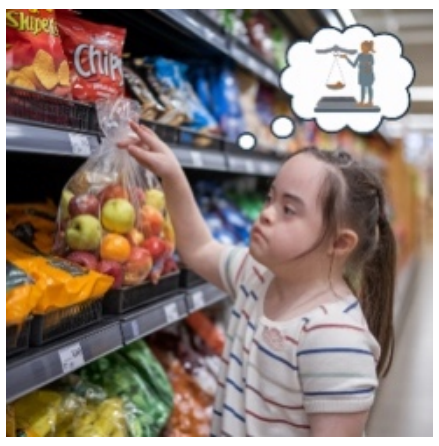	**4.5 I don't eat some kinds of food because they can make me fat.**	No	Maybe	Yes
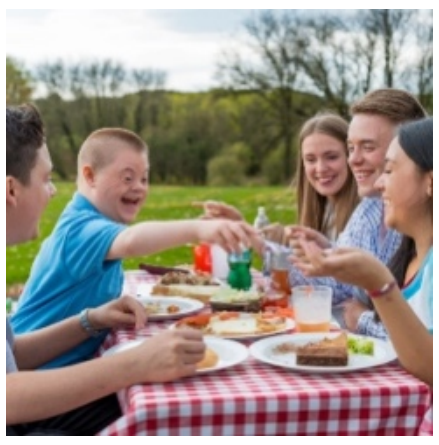	**4.6 When I am next to someone who is eating, I also feel like eating.**	No	Maybe	Yes
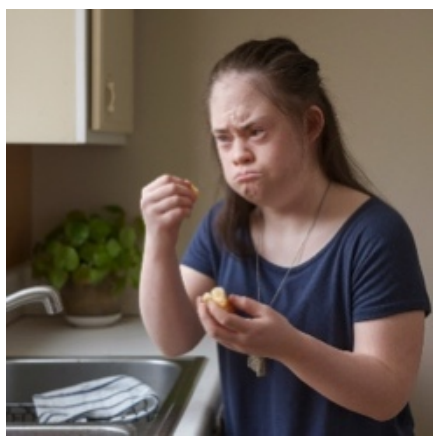	**4.7 When I feel angry, I need to eat.**	No	Maybe	Yes
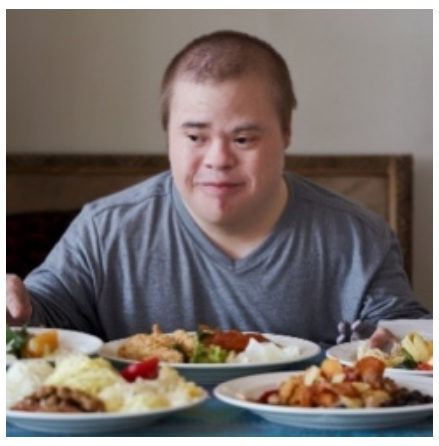	**4.8 I often get so hungry that I feel like I could eat loads of food without getting full.**	No	Maybe	Yes
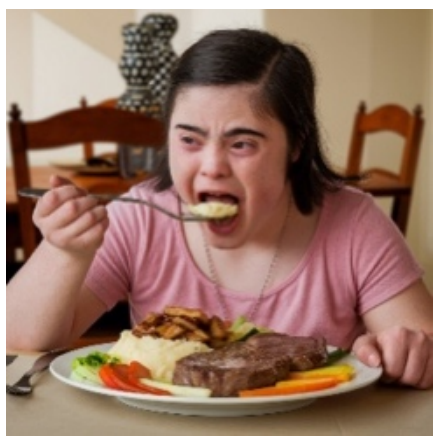	**4.9 When I am hungry, I feel like to have to eat all of the food on my plate in one go, without stopping.**	No	Maybe	Yes
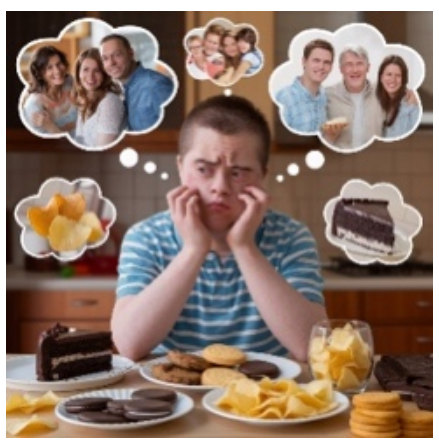	**4.10 When I feel lonely, I make myself better by eating.**	No	Maybe	Yes

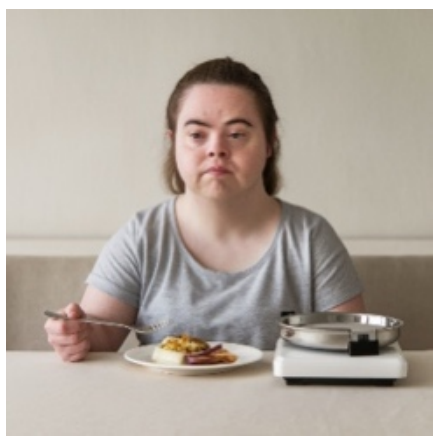	**4.11 I eat less than I want at meal times to stop myself putting on weight.**	No	Maybe	Yes
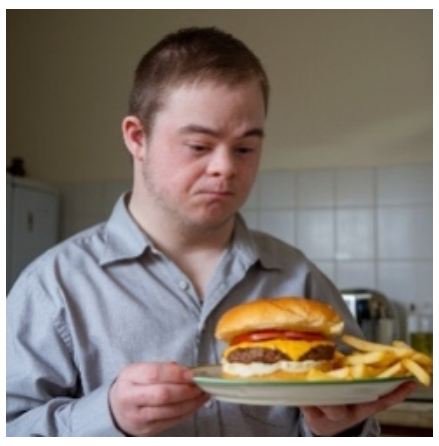	**4.12 When I smell or see my favourite food, I find it hard to stop myself from eating it, even if I've just finished a meal.**	No	Maybe	Yes
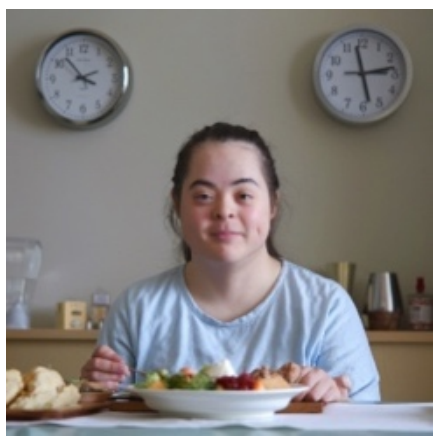	**4.13 I am always hungry enough to eat at any time.**	No	Maybe	Yes
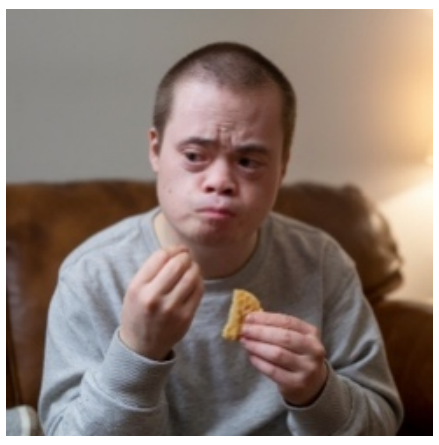	**4.14 If I feel nervous, I try to calm myself down by eating.**	No	Maybe	Yes
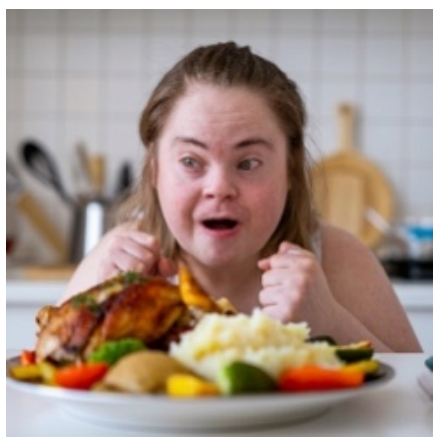	**4.15 When I see something that looks delicious, I get so hungry that I have to eat it right away.**	No	Maybe	Yes
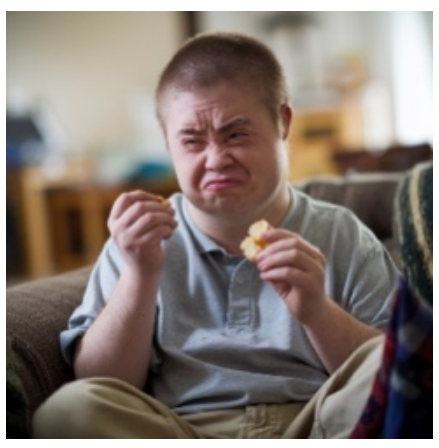	**4.17 When I feel really upset, I want to eat.**	No	Maybe	Yes
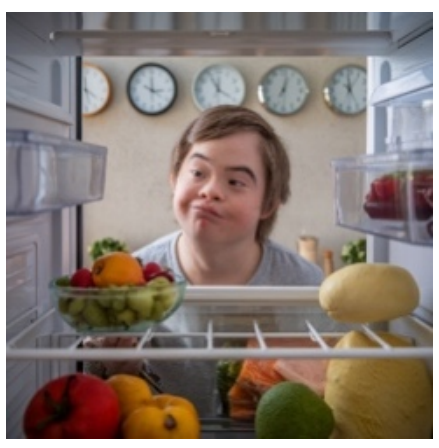	**4.18 How often do you feel hungry?**	Only at mealtimes	In between meals	All the time

5. Which do you prefer?

**5.1**	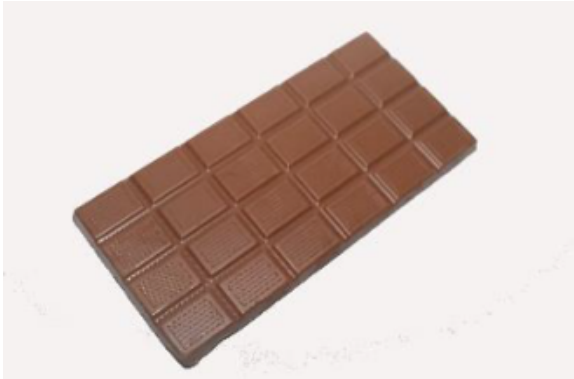 Chocolate bar	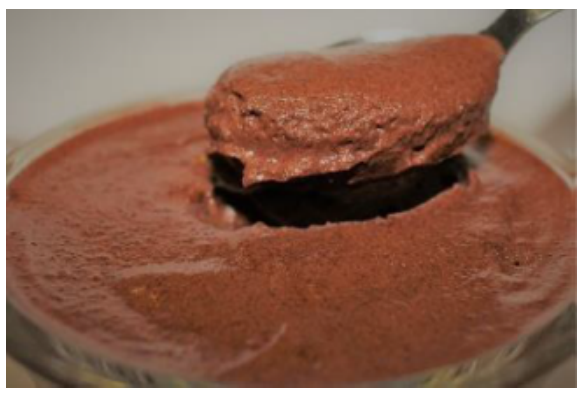 Chocolate mousse	Never tasted one or both
**5.2**	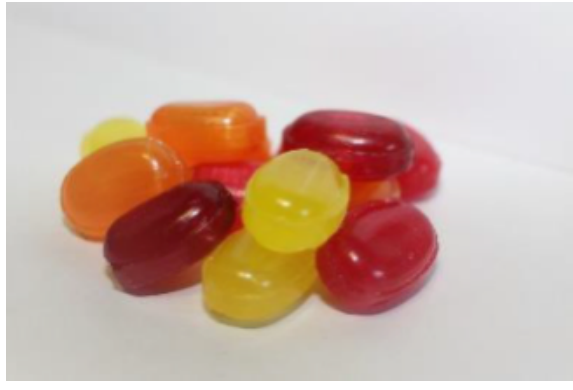 Boiled sweets	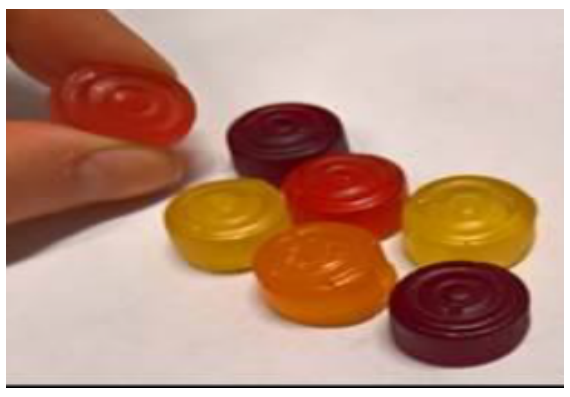 Chewy sweets	Never tasted one or both
**5.3**	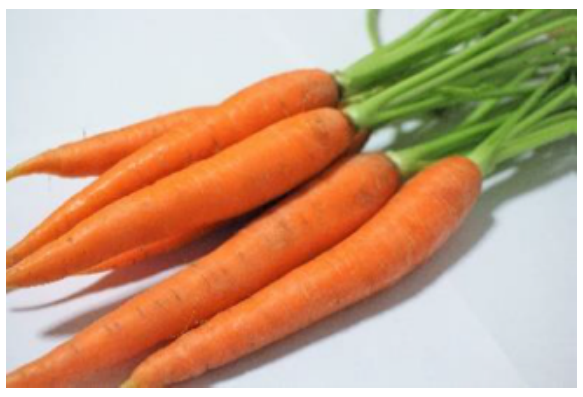 Raw carrot	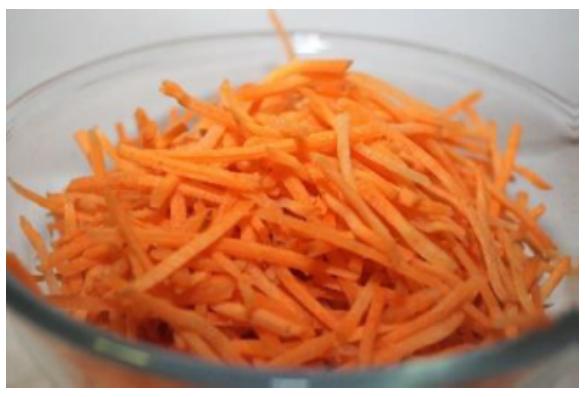 Grated carrot	Never tasted one or both
**5.4**	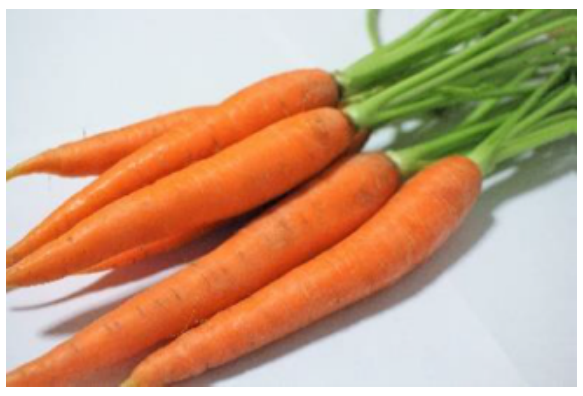 Raw carrot	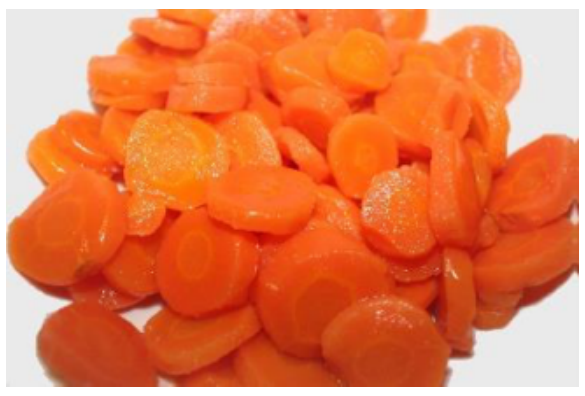 Cooked carro	Never tasted one or both
**5.5**	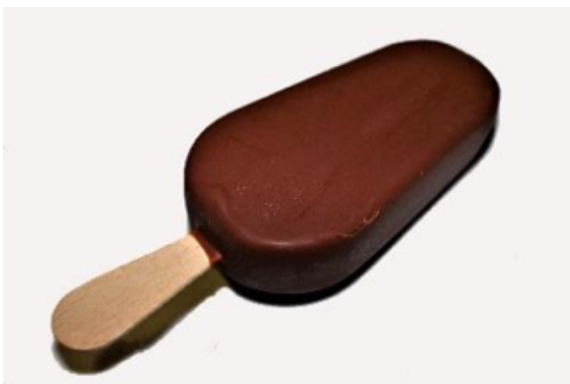 Crunchy Ice Cream	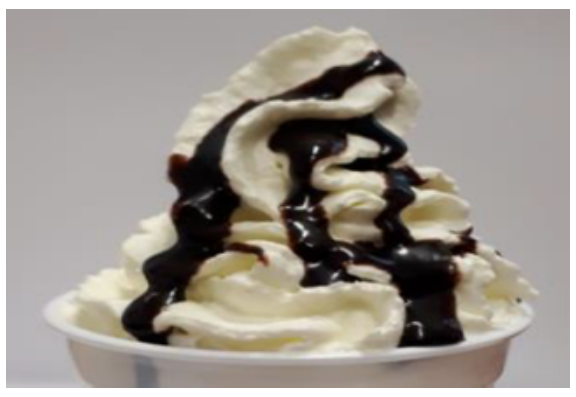 Soft Ice Cream	Never tasted one or both
**5.6**	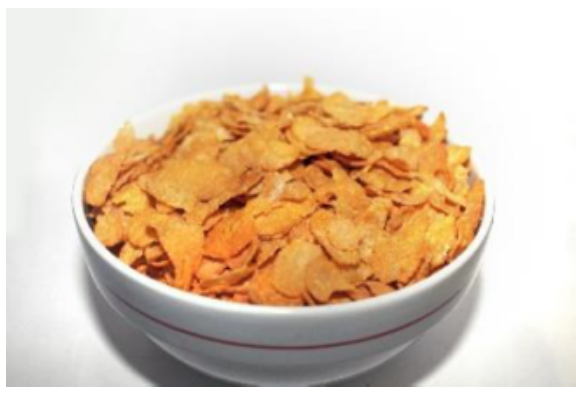 Corn Flakes	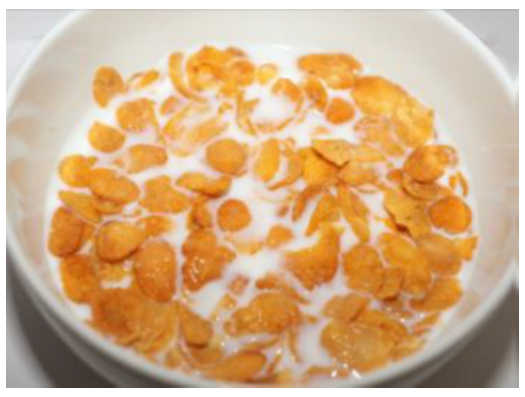 Soaked Corn Flakes (in Milk)	Never tasted one or both
**5.7**	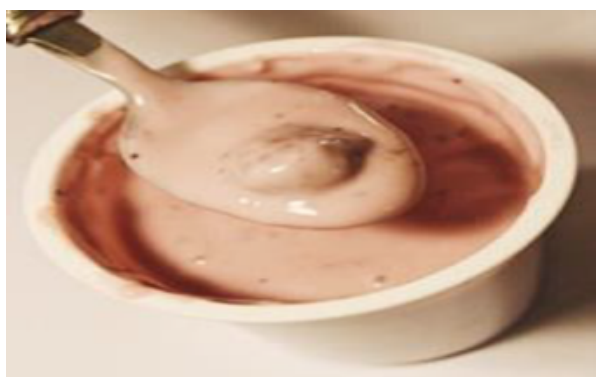 Yogurt with fruit pieces	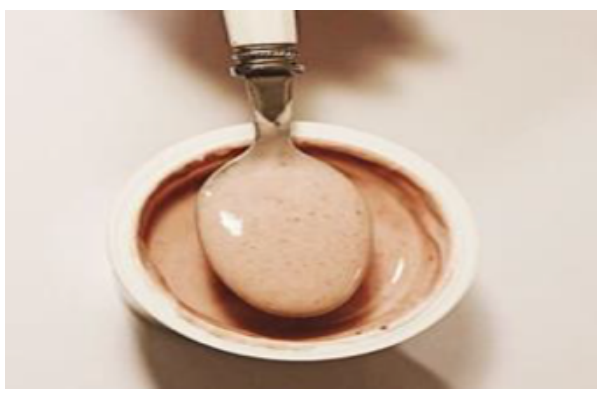 Yogurt without fruit pieces	Never tasted one or both

**5.8**	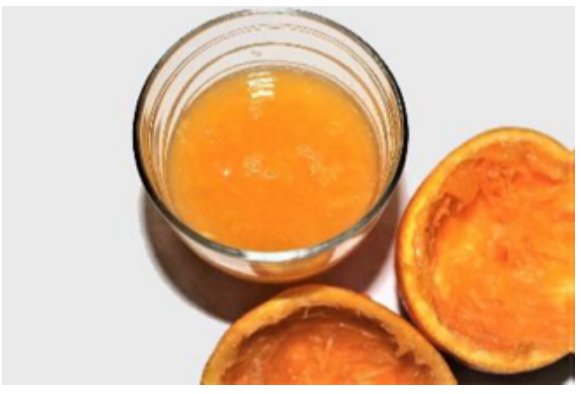 Orange juice with bits	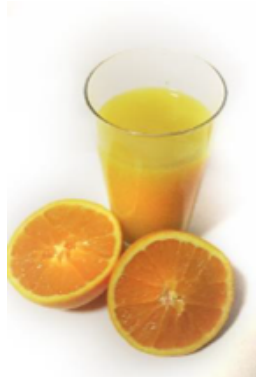 Smooth orange juice	Never tasted one or both
**5.9**	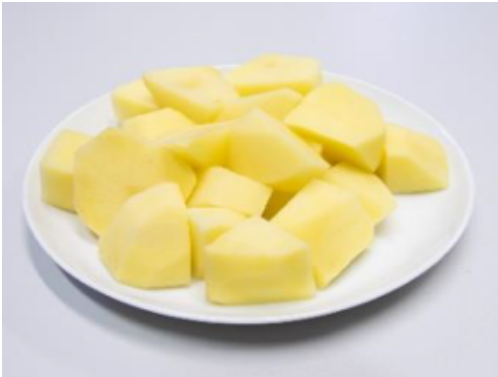 Boiled potatoes	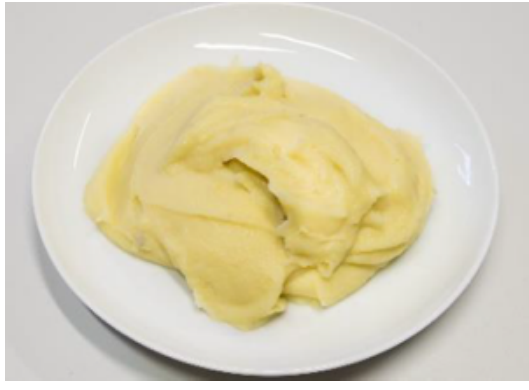 Mashed potatoes	Never tasted one or both
**5.10**	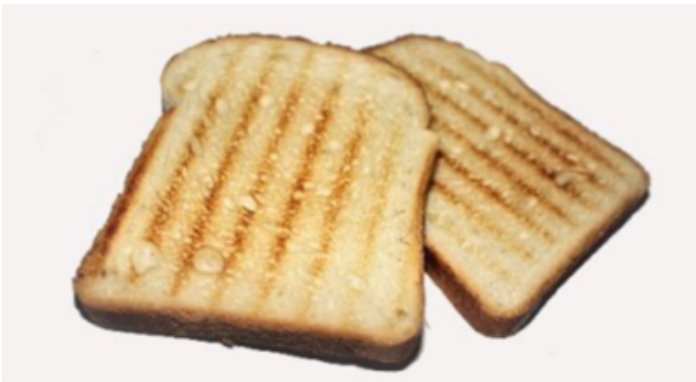 Toasted bread	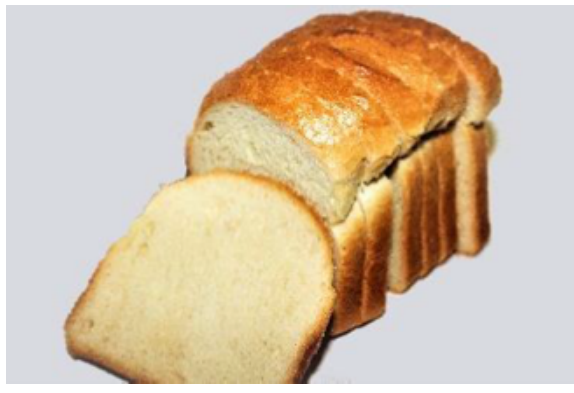 Bread	Never tasted one or both
**5.11**	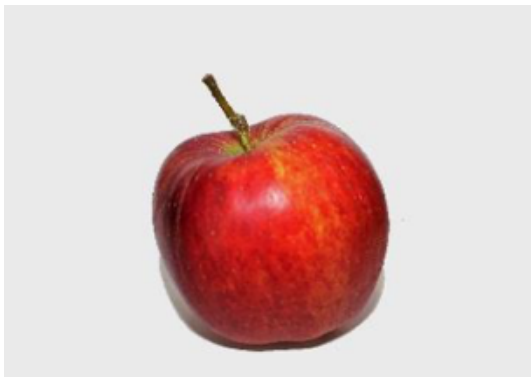 Apple	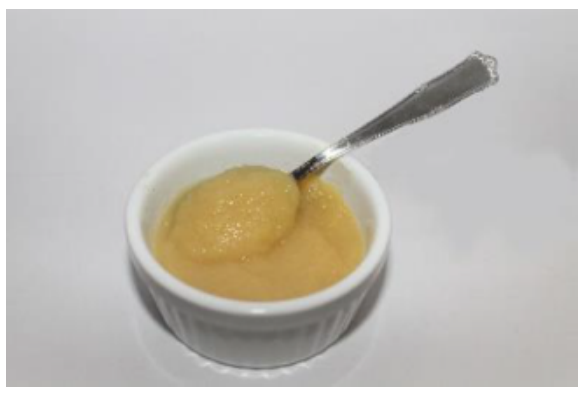 Apple puree	Never tasted one or both
**5.12**	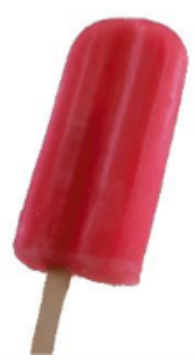 Ice lolly	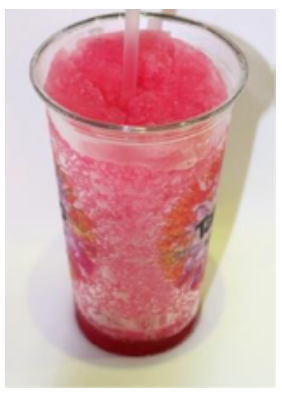 Sorbet/slushy	Never tasted one or both
**5.13**	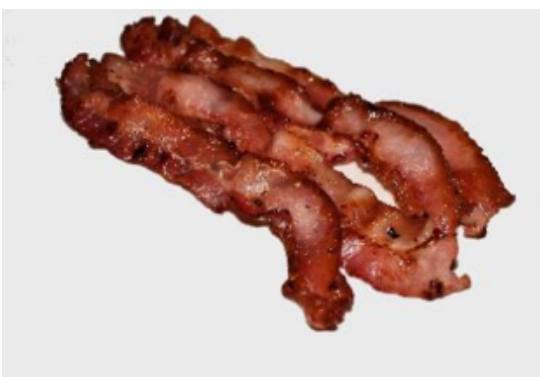 Crispy bacon	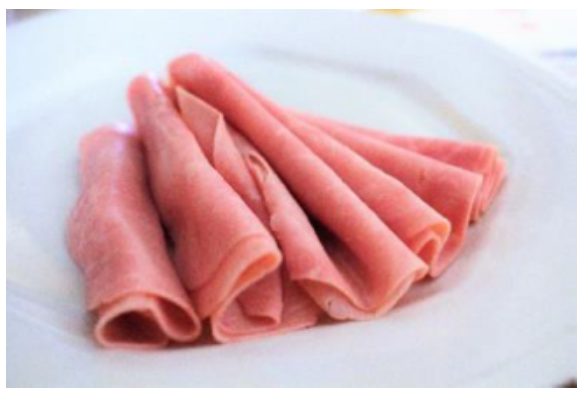 Ham	Never tasted one or both
**5.14**	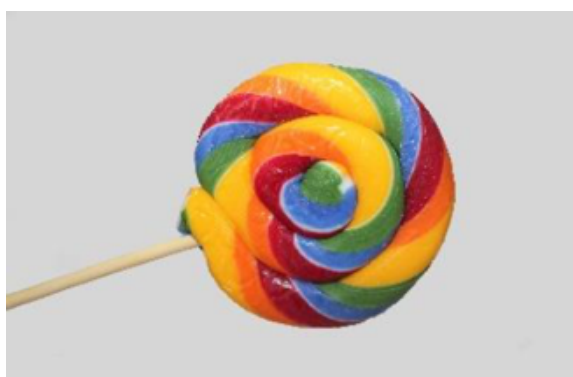 Lollipop	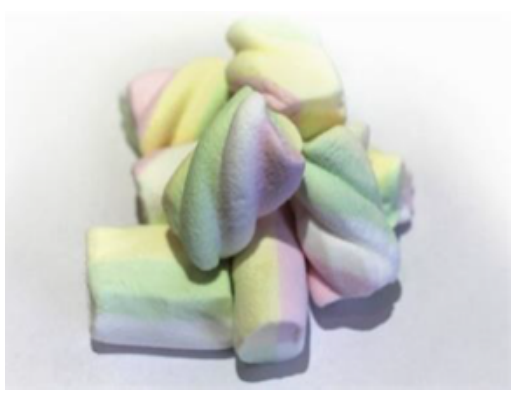 Marshmellow	Never tasted one or both

**5.15**	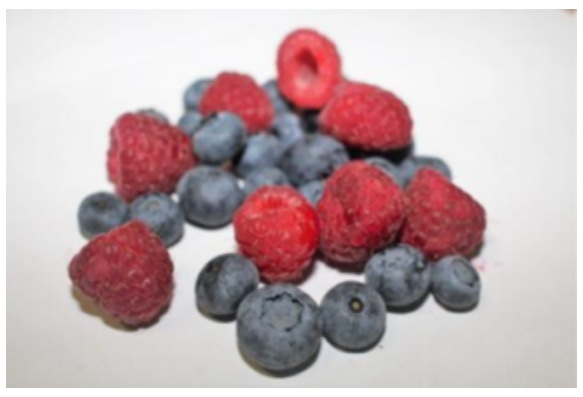<br>Berries	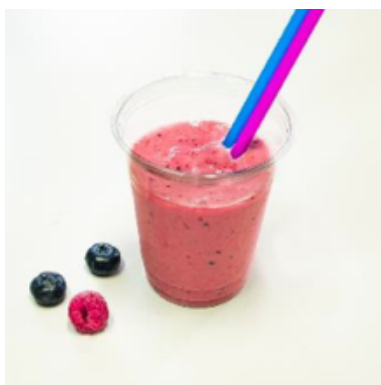<br>Berrie smoothie	Never tasted one or both
**5.16**	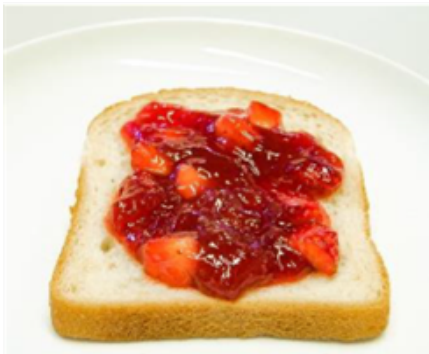<br>Jam with fruit pieces	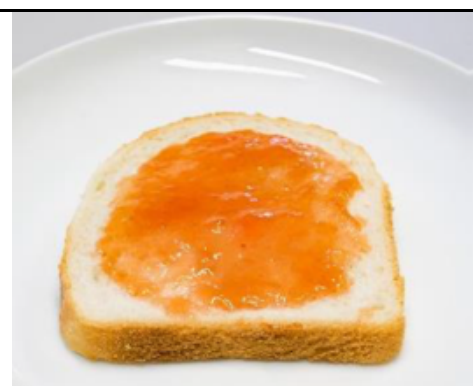<br>Jam without fruit pieces	Never tasted one or both
**5.17**	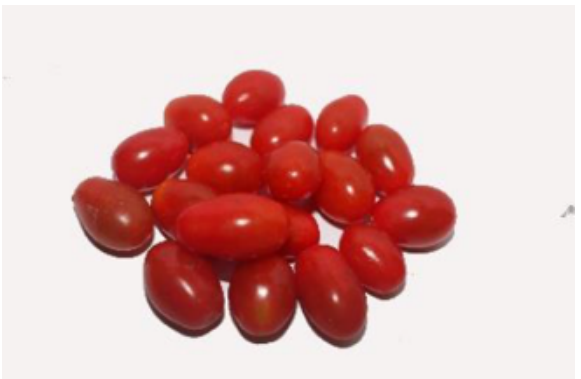<br>Tomatoes (cherry tomatoes)	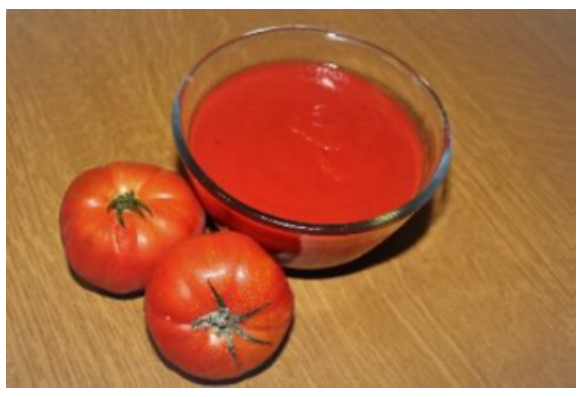<br>Tomato puree	Never tasted one or both

6. How true are these statements for you?

**Indicate how true each statement is for you:**				
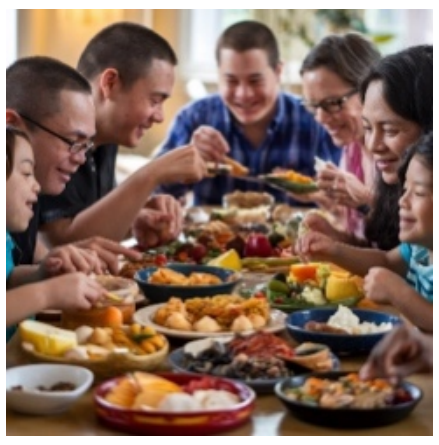	**6.1 Almost every day I eat new and unusual foods.**	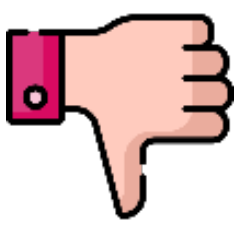<br>No	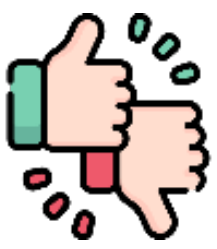<br>Maybe	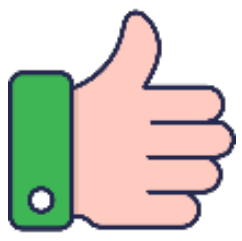<br>Yes
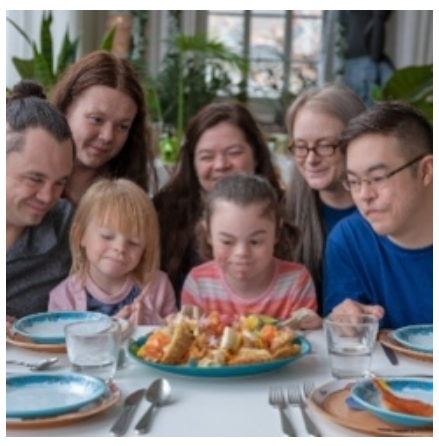	**6.2 I don't trust new foods**	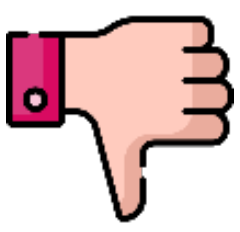<br>No	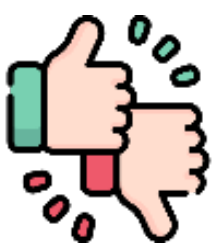<br>Maybe	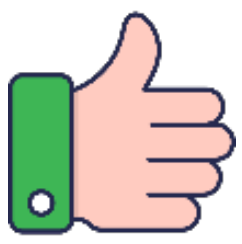<br>Yes
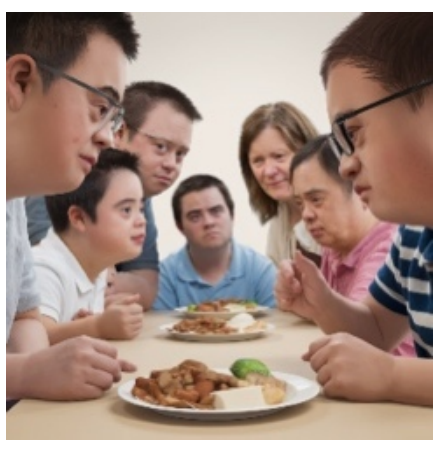	**6.3 If a food is new, I don't try it.**	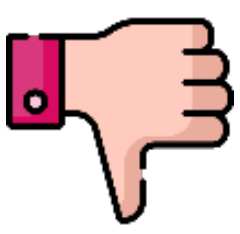<br>No	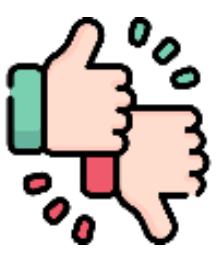<br>Maybe	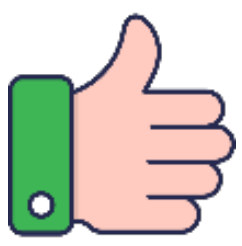<br>Yes

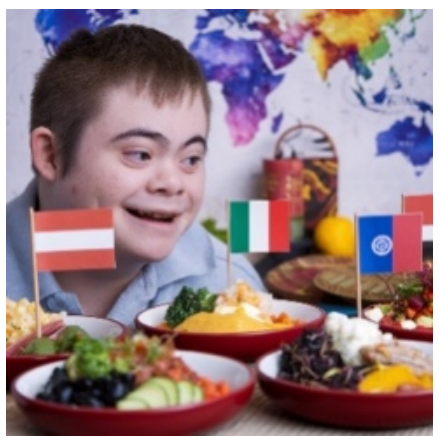	**6.4 I like to try tastes and foods which are unusual and come from different countries.**	No	Maybe	Yes
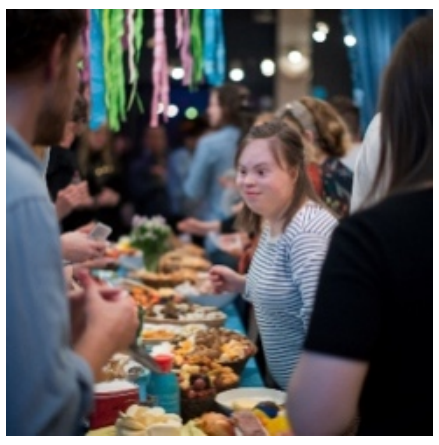	**6.5 When I am at a friend's party, I like to try new foods.**	No	Maybe	Yes
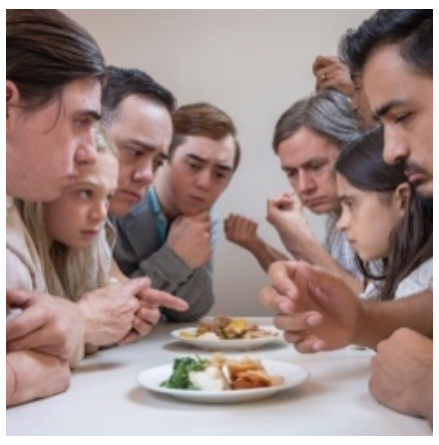	**6.6 I am afraid to eat foods I have never had before.**	No	Maybe	Yes
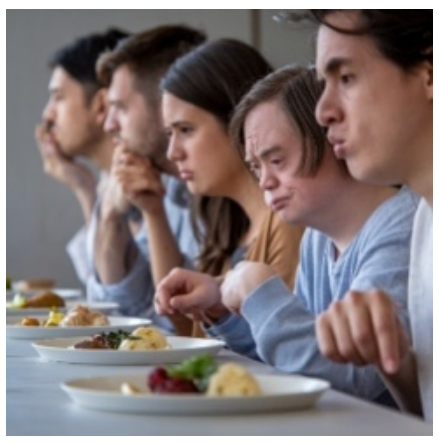	**6.7 I am very fuzzy about food.**	No	Maybe	Yes
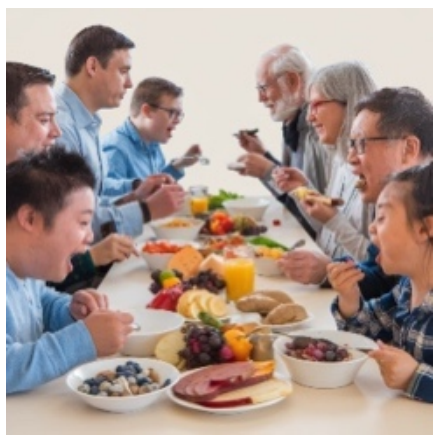	**6.8 I really eat everything!**	No	Maybe	Yes

7. The Environment

**Indicate how much you agree with each statement:**				
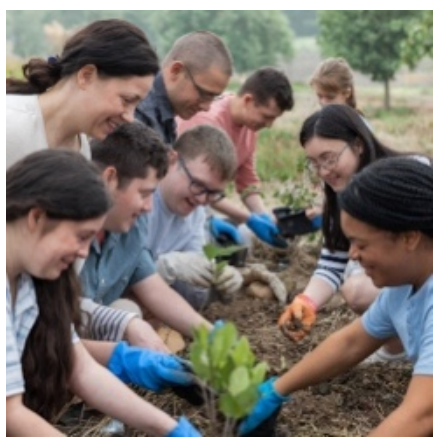	**7.1 I carry out activities to protect the environment (*Optional*)**	No	Maybe	Yes
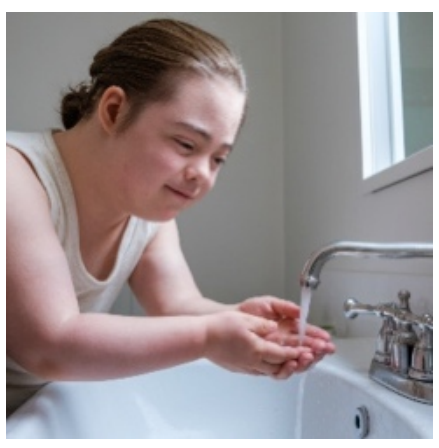	**7.2 I'm careful with the quantity of water I use when I take a shower or a bath**	No	Maybe	Yes

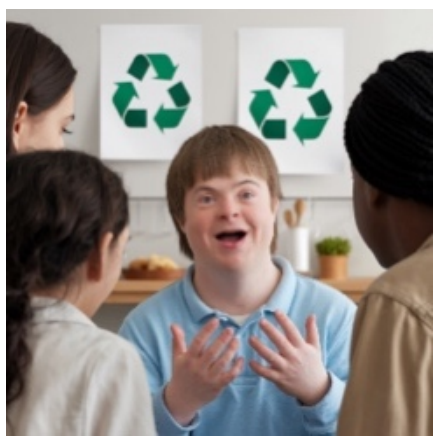	**7.3 I talk to my family, friends and peers about environmental protection (e.g., recycling)**	No	Maybe	Yes
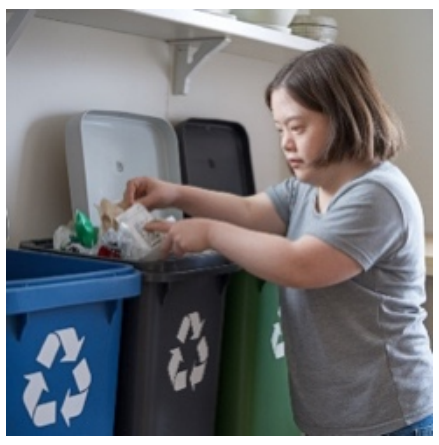	**7.4 At home, I help separate and to recycle.**	No	Maybe	Yes
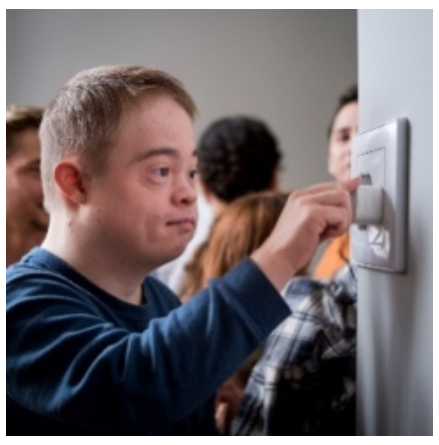	**7.5 I switch off the electrical appliances to save energy when I am not using them.**	No	Maybe	Yes

Submit your survey.
Thank you for completing this survey.
